# Novel Feather Degrading Keratinases from *Bacillus cereus* Group: Biochemical, Genetic and Bioinformatics Analysis

**DOI:** 10.3390/microorganisms10010093

**Published:** 2022-01-01

**Authors:** Arwa Ali Almahasheer, Amal Mahmoud, Hesham El-Komy, Amany I. Alqosaibi, Sultan Aktar, Sayed AbdulAzeez, J. Francis Borgio

**Affiliations:** 1 Department of Biology, College of Science, Imam Abdulrahman Bin Faisal University, P.O. Box 1982, Dammam 31441, Saudi Arabia; 2200500136@iau.edu.sa (A.A.A.); hmabdalla@iau.edu.sa (H.E.-K.); amgosaibi@iau.edu.sa (A.I.A.); 2 Basic & Applied Scientific Research Center, Imam Abdulrahman Bin Faisal University, P.O. Box 1982, Dammam 31441, Saudi Arabia; 3 Department of Biophysics, Institute for Research & Medical Consultations (IRMC), Imam Abdulrahman Bin Faisal University, P.O. Box 1982, Dammam 31441, Saudi Arabia; suakhtar@iau.edu.sa; 4 Department of Genetic Research, Institute for Research & Medical Consultations (IRMC), Imam Abdulrahman Bin Faisal University, P.O. Box 1982, Dammam 31441, Saudi Arabia; asayed@iau.edu.sa (S.A.); fbalexander@iau.edu.sa (J.F.B.)

**Keywords:** keratinase, chicken feather, biodegradation, *Bacillus cereus* group, subtilisin-like serine proteases, molecular docking

## Abstract

In this study, five keratinolytic bacteria were isolated from poultry farm waste of Eastern Province, Saudi Arabia. The highest keratinase activity was obtained at 40–45 °C, pH 8–9, feather concentration 0.5–1%, and using white chicken feather as keratin substrate for 72 h. Enhancement of keratinase activity through physical mutagen UV radiation and/or chemical mutagen ethyl methanesulfonate (EMS) resulted in five mutants with 1.51–3.73-fold increased activity over the wild type. When compared with the wild type, scanning electron microscopy validated the mutants’ effectiveness in feather degradation. Bacterial isolates are classified as members of the S8 family peptidase *Bacillus cereus* group based on sequence analysis of the *16S rRNA* and keratinase genes. Interestingly, keratinase *KerS* gene shared 95.5–100% identity to keratinase, thermitase alkaline serine protease, and thermophilic serine protease of the *B. cereus* group. D_137_N substitution was observed in the keratinase *KerS* gene of the mutant strain S13 (*KerS*13uv+ems), and also seven substitution variations in *KerS*26 and *KerS*26uv of strain S26 and its mutant S26uv. Functional analysis revealed that the subtilisin-like serine protease domain containing the Asp/His/Ser catalytic triad of *KerS* gene was not affected by the predicted substitutions. Prediction of physicochemical properties of *KerS* gene showed instability index between 17.5–19.3 and aliphatic index between 74.7–75.7, which imply keratinase stability and significant thermostability. The docking studies revealed the impact of substitutions on the superimposed structure and an increase in binding of mutant D_137_N of *KerS*13uv+ems (affinity: −7.17; S score: −6.54 kcal/mol) and seven mutants of *KerS*26uv (affinity: −7.43; S score: −7.17 kcal/mol) compared to the wild predicted structure (affinity: −6.57; S score: −6.68 kcal/mol). Together, the keratinolytic activity, similarity to thermostable keratinases, and binding affinity suggest that keratinases *KerS*13uv+ems and *KerS*26uv could be used for feather processing in the industry.

## 1. Introduction

Feathers are considered a byproduct of poultry production; untreated feather waste is a source of many pathogenic microorganisms and pollutants [1]. Chicken feather contains 90% keratin which is highly disulfide-bonded and resistant to degradation when treated with various proteases such as papain, pepsin, and trypsin [2,3,4]. Despite their rigid structure, keratinase degrades feather keratin more efficiently than other proteases [4,5]. 

Different *Bacillus* species are characterized as keratinase producers, i.e., *Bacillus licheniformis*, *B. megaterium*, *B. subtilis*, *B. cereus*, and *B. pumilus* [5]. Keratinases belong to the subtilisin group, serine protease (S8 family) [6,7]. In silico analysis was used to identify various bacterial keratinases. The sequence of the 27 N-terminal residues of *KERUS* shared a high degree of homology with those of *Bacillus* keratinases [8]. The keratinase gene (*dgeKer*) was identified from the *Deinococcus geothermalis* genome [9]. Amino acid sequences alignment indicated that the two keratinases of *B. pumilus* strain C4 are subtilisin-like serine proteases belonging to the protease S8 family [10].

Due to microbes’ keratinolytic enzymes in the feed, pharmaceutical industries, leather, fertilizer, and biological control in agriculture [11,12], mutagenesis has been used to enhance the overproduction of industrial strains which play a major role in the application of economically functional processes. New techniques such as genetic recombination have been studied to improve keratinase production, as well as improve its activity. However, mutagenesis and selection (i.e., random screening) is the frequent method of choice as it is a simple and cost-effective technique for the enhancement of industrial strains [13]. Chemical mutations using ethyl methanesulfonate (EMS) for *B. subtilis* resulted in mutant strains observed with 1.4–2.4-fold higher keratinolytic activity than the wild type [14]. Similarly, the mutant strain of *D. ficus* CC-ZG207 by UV resulted in a two-fold increase in the production of keratinase compared with the wild strain [15]. Gamma radiation mutagenic study increased keratinase production from 40.0 U/g for wild type to 120 U/g for the mutant *Penicillium chrysogenum* NNRL 792 [16].

It is worthy to note that keratinases have suitable tendencies for green technology. Therefore, continuance in the search for novel bacterial strains with keratinolytic activity and investigating their keratinolytic ability from diverse environments is imperative. Thus, this study was aimed to screen, isolate, and identify efficient keratinolytic bacterial isolates, as well as to increase keratinase production through physical and chemical mutagenesis. In addition, the keratinolytic bacteria and their keratinase were identified *in silico;* keratinase sequence and structure analysis was used to determine the effect of mutagenic treatments on the keratinase gene.

## 2. Materials and Methods

### 2.1. Bacterial Isolation, Screening for the Keratinolytic Activity, and Keratinase Assay

Samples of poultry farm wastes, feathers, and soil were collected from 5–6 cm depth from several poultry farms located in Dammam city, AL-Qatif city, Al-Ahsa city, and Al-Jubail city, Eastern Province, Saudi Arabia.

Bacterial isolation was carried out according to Subugade et al. [17]. Bacterial isolates from feathers were isolated by plating a portion of the feathers on a nutrient agar medium. The soil bacteria were isolated by serial dilution up to 10^−3^. Plates were incubated at 30 °C for 24 h. The bacterial isolates were further subcultured to obtain a pure culture. The purity of the gram-stained bacteria was observed under a high-power magnifying lens (100×) in a light microscope [17]. The bacterial isolates were maintained on nutrient agar slants and stored at 4 °C.

Screening for proteolytic activity was carried out according to Reyes et al. [18] using the following medium: 5 g/L peptone, 3 g/L yeast extract, 100 mL/L sterile non-fat milk, and 20 g/L agar. Bacteria were incubated at 30 °C for 72 h. Isolates that depicted the highest hydrolytic zone of clearance around its colonies in this medium were selected. The processing of feathers was carried out according to Rajak et al. [19]. White chicken feathers were washed with warm water and detergent, followed by defatting and surface sterilized with petroleum-ether aliquots for over 24 h. Finally, they were washed with distilled water and dried in an oven at 50 °C for 24 h.

The method of Aly et al. [20] was used to evaluate the keratinolytic properties of the tested bacteria as follows: One hundred ml Erlenmeyer flasks containing 50 mg washed defatted white chicken feathers in 20 mL growth basal salt medium composed of (g/L) NH_4_Cl 0.5 g; NaCl 0.5 g; KH_2_PO_4_ 0.3 g; K_2_HPO_4_ 0.3 g; yeast extract 0.1 g; MgCl_2_·6H_2_O, 0.1 g and pH was adjusted to 7. The autoclaved medium was inoculated with 1 mL bacterial cell suspension (1 mL of 24 h old bacterial suspension grown in 5 mL of the nutrient broth medium at 30 °C). The cultures were incubated at 37 °C under shaking at 270 rpm for 72 h. Keratinase activity of culture filtrate was assayed using a modified protocol of Preczeski et al. [21] with keratin azure as a substrate. The reaction mixture contained 0.4 mL of crude enzymes and 1.6 mL of 0.4% keratin azure (Sigma K8500, Saint Louis, MI, USA) in 10 mM tris HCl (pH 8.5) buffer incubated at 50 °C for 1 h. Subsequently, the reaction was stopped with 0.8 mL of 10% trichloroacetic acid (TCA) then centrifuged at 5000 rpm for 20 min. A control sample was prepared in a similar manner except that the bacteria were replaced by the same volume of dH_2_O. Unit of keratinase activity was defined as a 0.01 unit increase in absorbance at 595 nm.

### 2.2. Factors Affecting Keratinase Activity

The effect of the incubation period on keratinase activity was determined according to Dhiva et al. [22]. Incubation period effects on enzyme activity were determined at 24 h intervals at 45 °C during an incubation period of 24–96 h at initial pH 7.

The effect of temperature on enzyme activity was analyzed in a varied temperature range (35 °C, 40 °C, 45 °C, 50 °C, and 55 °C) at initial pH 7 [20].

The effect of the initial pH of the medium on keratinase activity was determined according to Aly et al. [20]. pH effects on the enzymatic activity were analyzed at 45 °C in varied initial pH values ranging from 6 to 9.

According to Kalaikumari et al. [2], the effect of supplementation of additional nitrogen source (NH_4_Cl), carbon source (glucose), and sulfur source (MgSO_4_·7H_2_O) individually and in combination with white chicken feathers on the keratinase activity was determined at initial pH 7.5 and 45 °C.

Moreover, the impact of white chicken feather concentrations on the enzymatic activity was assessed at 45 °C in a varied feathers concentration range (0.5%, 1%, 1.5%), and 2% at initial pH 7.5 [2].

### 2.3. Feather Biodegradation In Vitro

The influence of keratin substrate on the enzymatic activity was investigated at 45 °C in a varied keratinaceous material (white chicken feather, black chicken feather, white sheep wool, black sheep wool, and hair) as carbon and energy source at initial pH 8 [23].

### 2.4. Random Mutagenesis

UV-induced mutagenesis was performed according to Aly and Tork, [24]. The wild-type bacteria (S1, S13, S15, S26, and S39) were cultured on nutrient agar plates at 30 °C for 24 h, then exposed to UV irradiation at 254 nm and 365 nm for 10 min, 20 min, and 30 min at distances of 10 cm and 20 cm from the UV lamp. Plates were incubated overnight at 30 °C. The keratinolytic activity of the mutant isolates was evaluated using skim milk plates as well as the previously described basal salt medium.

Ethyl methanesulfonate (EMS)-induced mutagenesis study was carried out according to the method of de Paiva et al. [14]. Wild bacterial isolates (S1, S13, S15, S26, and S39), and UV mutated isolates (S13uv and S26uv) were grown in 5 mL of nutrient broth medium at 30 °C for 24 h; after that, 1% of EMS was added and incubated at 30 °C for 2 h. Cells were then centrifuged at 5000 rpm for 7 min, washed twice with sterile distilled water and the pellet was resuspended in 5 mL of nutrient broth medium and incubated at 30 °C for 1 h. Successive serial dilutions were prepared up to 10^−3^, and 0.1 mL of the bacterial dilutions were spread on a nutrient agar medium. The keratinolytic activity of the mutant isolates was tested using skim milk plates as well as the previously described basal salt medium.

### 2.5. Evaluation of Biodegradation Efficiency of the Wild and Mutant Isolates by Scanning Electron Microscopy

To check for keratinase activity, the structural changes of biodegraded feathers were examined by scan electron microscopy (SEM) as described by Gupta and Singh [25]. Degraded chicken feathers after 72 h of incubation with keratinolytic bacterial isolates S13, and S39, and their mutants were recovered and oven-dried at 50 °C. After slicing into 1 cm, the samples were sterilized with 70% ethanol for 10 min, then fixed with 2.5% glutaraldehyde for 4 h and washed with distilled H_2_O for 5 min. Samples were dehydrated by a series of ethanol (30%, 50%, 70%, 90%, and 100% for 10 min) at room temperature, followed by critical point drying. Later, feathers samples were sputter-coated with gold and observed by SEM (FEI, Inspect S50, Brno, Czech Republic) at an accelerating voltage of 20 kV.

### 2.6. Feather Hydrolysis Assay

The degree of feather hydrolysis by the tested bacteria was assessed according to the weight-loss method of Nnolim et al. [26]. The fermentation broth was filtered (Whatman^®^ qualitative filter paper, Grade 1, Maidstone, UK) to recover undegraded feathers, and oven dried at 50 °C for 24 h, and the constant weight was achieved. The degree of feather hydrolysis was calculated as shown in Equation (1):

(1) $$\%\;of\;hydrolysis=\left(\frac{\mathrm{IM}-\mathrm{FM}}{\mathrm{IM}}\right)\times 100$$

where, (IM) is the initial dry mass of the intact feather before the fermentation process, and (FM) is the dry mass of the residual feather after the fermentation process.

### 2.7. Statistical Analysis

All in vitro experiments were performed in duplicate. Data obtained were analyzed by ANOVA test and means were compared by Duncan’s (SPSS 22.0 version). Differences were considered significant when *p* < 0.05. Values are expressed as means ± standard error (SE). Mean with the different letters are significantly different [27].

### 2.8. PCR Amplification of 16S rRNA and Keratinase Genes

The *16S rRNA* gene of the keratinolytic isolates (S1, S13, S15, S26, and S39) was amplified using colony PCR and the following primers: Forward 5′-AGAGTTTGATCCTGGCTCAG-3′ and reverse 5′-TACGGCTACCTTGTTACGACTT-3’), (Applied Biosystems, Foster City, CA, USA). The PCR reaction was carried out using PCR master mix (MOLEQULE-ON, Auckland, New Zealand) in Biometra T-Professional thermocycler (Biometra, Goettingen, Germany) with an annealing temperature of 56 °C for 35 cycles [28].

Keratinase (*KerS*) gene was amplified from wild-type isolates (S1, S13, S15, S26, and S39) and their mutants (S1ems, S13uv, S13uv+ems, S15ems, S26uv, and S39ems). Bacterial colonies were used for direct amplification of the keratinase gene. Keratinase primers were designed using *Bacillus cereus* strain BHU2 chromosome (CP023726.1): BaCeKerF 5′ATYGAGAATCCATATGTAGGAAAATTAG-3′ and BaCeKerR 5′CATCCCCTCTTTTACTTWATTACTATCAT-3′ for the amplification of the entire gene (1660 bp).

PCR amplification of *KerS* gene was performed using PCR master mix (MOLEQULE-ON, Auckland, New Zealand) in Biometra T-Professional thermocycler (Biometra; Goettingen, Germany) with the annealing temperature at 54 °C for 35 cycles. The PCR amplicons were visualized using 2% agarose gel electrophoresis and purified using QIAquick PCR Purification Kit (Qiagen, Hilden, Germany). The purified products of *16S rRNA* and keratinase genes were sequenced with the same forward reverse primers using 3500 genetic analyzers (Applied Biosystems, Foster City, CA, USA) through BigDye^®^ Terminator v3.1 Cycle Sequencing Kit (Applied Biosystems, Foster City, CA, USA).

### 2.9. Sequence Similarity Search and GenBank Submission

Sequences were checked and edited using FinchTV (https://finchtv.software.informer.com/1.4/, accessed on 20 December 2021). The sequence was analyzed using the BLAST program (http://www.ncbi.nlm.nih.gov/blast, accessed on 20 December 2021). Sequences were submitted to GenBank (https://www-ncbi-nlm-nih-gov.library.iau.edu.sa/WebSub/, accessed on 20 December 2021) under the accession number OL441832-OL441836 and OL448296-OL448306 for the *16S rRNA* and keratinase gene sequences, respectively.

### 2.10. Phylogenetic Analysis of 16S rRNA and Keratinase Genes

For sequence comparison, *16S rRNA* and keratinase sequences were retrieved from the National Center for Biotechnology Information (NCBI) database (http://www.ncbi.nlm.nih.gov, accessed on 20 December 2021). Sequence alignment analysis and phylogenetic tree construction were performed by MEGA 6.0.

### 2.11. Functional Analysis of Keratinase Gene

Expasy-PROSITE tools are protein databases for identifying protein domains, families, and functional sites as well as associated patterns and profiles [29]. ScanProsite, one of the Expasy-PROSITE tools, was used to predict the catalytic domain and the active sites of the *KerS* gene.

### 2.12. Physicochemical Characterization of Keratinase Gene

The physical and chemical attributes, such as molecular weight, theoretical isoelectric point (*pI*), amino acid composition, instability index, aliphatic index, and grand average of hydropathy (GRAVY) were computed using the ProtParam assessment tool of the ExPASy server (http://web.expasy.org/protparam/, accessed on 20 December 2021).

### 2.13. Structure Modeling and Analysis of Wild-Type Keratinase and Mutants

The Swiss MODEL server was used to predict the structural modeling of keratinase *KerS* protein, and to create mutated keratinase, *KerS*13uv+ems (D_137_N), and the 7 substitutions (N_117_K, V_195_I, A290G, S_295_L, R_297_K, T_364_S, and S_368_T) that differentiated between *KerS*26uv and the other 4 keratinase strains. Visualization of the modeled PDB was done using PYMOL and validated using PROCHECK. Ramachandran plot statistics using the PDBsum structural analysis server were used to validate the 3D models. The suitable model for keratinase protein was selected based on the criteria of having the highest number of amino acid residues in the most favored region and the minimum number of residues in the outlier region, and the same was used for further analysis. In the validated model, 3D atomic coordinates of the receptor were used to verify potential sites for binding of substrate docking [30].

### 2.14. Molecular Docking Study of Keratinase KerS Gene

Docking of the keratinase protein modeled structures of wild and mutant types was performed separately with *N*-succinyl-l-alanyl-l-alanyl-l-prolyl-l-phenylalanine 4-nitroanilide (S-9205) as a substrate to analyze the substrate specificity and active site analysis. The modeled structures were 3D protonated, and then docking was performed with the selected ligand *N*-succinyl-l-alanyl-l-alanyl-l-prolyl-l-phenylalanine 4-nitroanilide. The settings of MOE software were rescoring1 at London dG and rescoring2 at GBVI/WSA dG, and the ligand interaction was performed with keratinase protein. Energy minimization was performed for both ligands and proteins [30].

## 3. Results and Discussion

### 3.1. Isolation and Screening of Keratinolytic Bacteria

**Figure 1:**
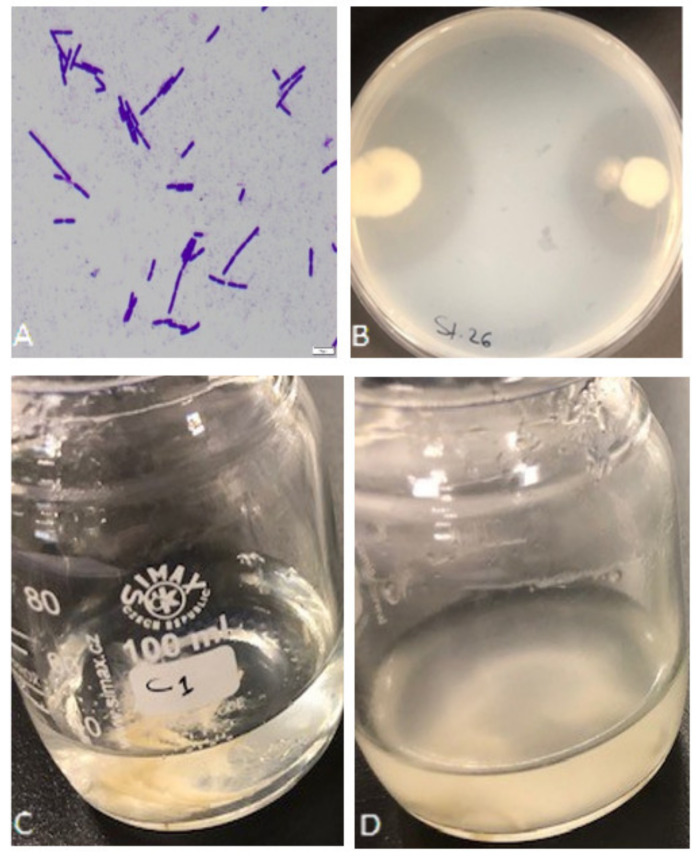
Isolation and screening of keratinolytic bacteria. (**A**) Bacterial isolates under the light microscope after gram staining. (**B**) Preliminary screening of isolate S26 for proteolytic activity on skim milk agar showing the formation of a clear zone around the colonies. (**C**) Growth medium before bacterium inoculation. (**D**) Feather degradation with bacterial isolate S1 after 72 h of shaking incubation at 37 °C.

A total of 42 bacterial isolates were isolated from different samples of poultry farm waste, in Eastern Province, Saudi Arabia. Based on the bacterial shape observed under the light microscope, microscopic examination of the new isolates revealed that the cells were rod-shaped, straight, occurring singly, in pairs, or chains, and gram-positive; 18 isolates were considered pure and chosen for further study (Figure 1A). Upon preliminary screening, the eighteen isolates showed proteolytic activity, forming a remarkable hydrolytic zone of clearance (20–38.5 mm) around their colonies confirming the degradation and utilization of skim milk (Figure 1B). Five of the high clearance zone isolates (S1, S13, S15, S26, and S39) were selected for further analysis. Keratinase production of the 5 isolates was determined as a second selection in the basal mineral media using feather as the sole carbon and nitrogen source (Figure 1C,D); isolates showed keratinase activity of 0.9–5.9 U/mL. The maximum keratinase activity of *Exiguobacterium* sp. and *S. radiopugnans* was 2.87 U/mL and 3.26 U/mL, respectively, in reported studies [24,27].

### 3.2. Effect of Different Factors on Keratinase Production

**Figure 2:**
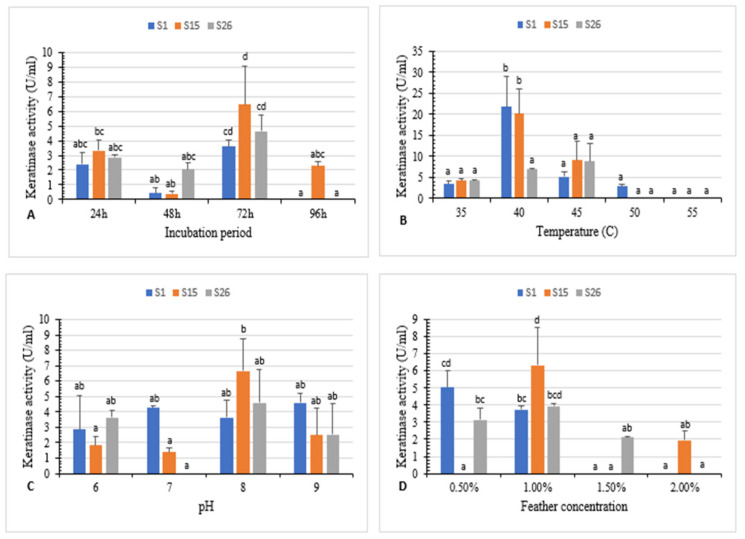
(**A**) Effect of incubation period, (**B**) temperature, (**C**) pH, and (**D**) feather concentration on white chicken feather degradation by isolates S1, S15, and S26. Bars are the standard error of the mean. Mean with the different letters are significantly different according to Duncan’s at *p* < 0.05.

Bacterial isolates S1, S15, and S26 were used to investigate the effect of the incubation period, temperature, pH, substrate concentration and different combinations of carbon, nitrogen, and sulfur (Figure 2 and Supplementary Materials Figure S1).

The maximum enzyme activity (3.6 U/mL, 6.4 U/mL, and 4.6 U/mL for isolates S1, S15, and S26, respectively) was attained at 72 h of incubation (Figure 2A). Previous research indicates that maximum keratinase activity was obtained after 48 h, 120 h, and 72 h of incubation for *Aeromonas hydrophila*, *Bacillus* sp., and *B. megaterium*, respectively [5,26,31].

The temperature effect on keratinase activity revealed that the highest keratinase activity (21.8 U/mL and 20.1 U/mL) was observed at 40 °C for S1 and S15, respectively, and 8.7 U/mL at 45 °C for S26. Further, an increase in temperature at 50 °C and 55 °C significantly reduced the activity (Figure 2B).

The effect of various initial pH values on keratinase activity was studied. Results revealed enzyme activity in a pH range of 6–9. The highest enzyme activity (6.6 U/mL and 4.6 U/mL) was observed at pH 8 for isolates S15 and S26, respectively, and (4.5 U/mL) at pH 9 for isolate S1 (Figure 2C).

Our results agree with Abdel-Naby et al. [32], who indicated that maximum enzyme activity for *B. cereus* PP/S6-3 was detected at pH 8 and 40 °C, and pH 9 and 50 °C for *B. cereus* PM/UM90. Likewise, a significant increase in keratinase activity of *S. radiopugnans* KR12 was noticed at pH 8 and 40 °C [24]. Martinez et al. [33] showed that keratinases isolated from various sources including, skin, feathers, hair, nails, soil, geothermal hot stream, and wastewater have an optimum range of pH 5.5 to pH 12.5 and temperature from 30 °C to 100 °C.

The effect of different concentrations of white chicken feather (0.5–2%) on keratinase activity was investigated. Maximum keratinase activity (6.3 U/mL and 3.9 U/mL) was obtained at 1% substrate concentration for isolate S15 and S26. However, 0.5% feather concentration was the optimum (5.0 U/mL) for isolate S1 after 72 h of incubation. (Figure 2D). Similarly, keratinase production was the highest at 0.5% and 1% substrate concentration for *Bacillus* sp. FPF-1 and *B. licheniformis* ALW1, respectively [13,26].

Supplementation of an additional MgSO_4_·7H_2_O as a sulfur source individually and in combination with NH_4_Cl as a nitrogen source increased keratinase activity of S1; the addition of NH_4_Cl individually increased keratinase activity of S26 and decreased keratinase activity of S15 when compared with the medium supplemented with feather only (control). However, the addition of glucose as a carbon source resulted in a decrease in keratinase activity (Supplementary Materials Figure S1). Chicken feather is made up of ß-keratin protein which is rich in both carbon and nitrogen. The addition of carbon, sulfur, and nitrogen sources along with feathers has been shown to improve the production of keratinase [34,35,36]. In contrast, results of Suntornsuk and Suntornsuk [37] found that glucose had no effect on keratin degradation with *Bacillus* sp. FK46 strain. Moreover, El-Refai et al. [38] reported that ammonium chloride did not promote keratinase production by *B. pumilus* FH9.

### 3.3. Feather Biodegradation In Vitro

**Figure 3:**
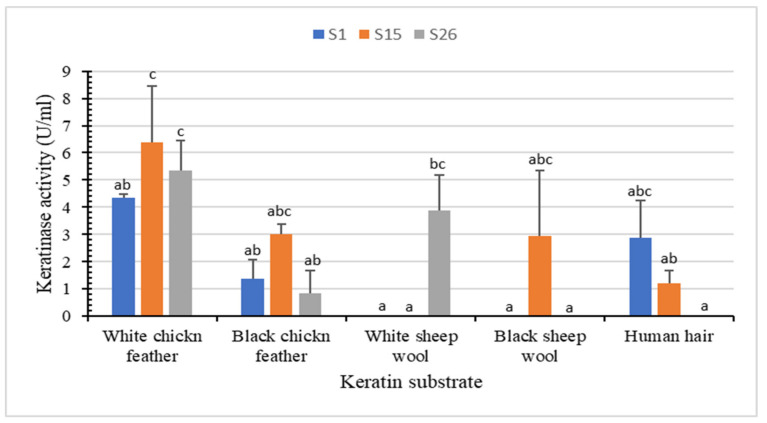
In vitro biodegradation of different keratin substrates (white chicken feather, black chicken feather, white sheep wool, black sheep wool, and human hair) after incubation with the keratinolytic bacteria for 72 h at 45 °C. Bars are the standard error of the mean. Mean with the different letters are significantly different according to Duncan’s at *p* < 0.05.

The keratin-biodegradation ability and keratinase activity by S1, S15, and S26 using various keratin substrates (white chicken feather, black chicken feather, white sheep wool, black sheep wool, and human hair) were studied (Figure 3 and Supplementary Materials Figure S2).

White chicken feather as substrate yielded the highest keratinase activity (6.3 U/mL for S15, 5.3 U/mL for S26 and 4.3 U/mL for S1), followed by white sheep wool (3.8 U/mL for S26), black chicken feather (3.0 U/mL for S15), black sheep wool (2.9 U/mL for S15) and human hair (2.8 U/mL for S1) (Figure 3). No degradation was observed with the control (Supplementary Materials Figure S2). Isolate S15 showed partial degradation of the white chicken feather (up to 52.50%) and produced the highest keratinase production (6.3 U/mL) at 45 °C and pH 8.85 after 72 h of incubation. Unlike other keratinolytic *Bacillus* spp. that demonstrated remarkable feather degradation after 7–10 days [39,40], significant partial degradation of white sheep wool, black chicken feather, and black sheep wool indicate the ability of our isolates to degrade both α-keratin and β-keratin. Similarly, *B. megaterium* F7-1 effectively degraded feather meal, duck feather, and human nail, whereas human hair and sheep wool showed relatively low degradation rates [39].

### 3.4. Improvement of Keratinase Production by Random Mutagenesis

The keratinase activity of the five keratinolytic bacterial isolates was developed by exposure to UV radiation and ethyl methanesulfonate (EMS), individually and in a combination of UV and EMS.

**Figure 4:**
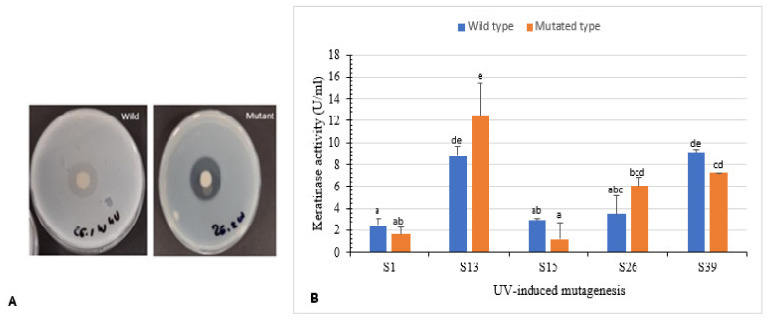
Effect of UV-random mutagenesis on keratinase activity. (**A**) Wild and UV-mutated isolate S26 showing clear zone of hydrolysis on skim milk agar. (**B**) Keratinolytic activity of wild isolates and their corresponding mutants. Bars are the standard error of the mean. Mean with the different letters are significantly different according to Duncan’s at *p* < 0.05.

Isolates S1, S13, S15, S26, and S39 were used to enhance keratinase production using UV radiation. Clear zone hydrolysis using skim milk for the wild and mutated isolates is shown in Figure 4A. Mutants S13uv and S26uv showed high keratinase activity (12.4 U/mL and 6.0 U/mL, respectively) compared with their wild isolates S13 and S26 (8.7 U/mL and 3.4 U/mL, respectively) after 72 h of incubation (Figure 4B). Zeng et al. [41] reported that mutant CC-ZG207 of *D. ficus* showed 2-fold higher keratinolytic activity after 10 days of incubation; in contrast, the activity of mutant CC-ZG227 was lower than wild type.

**Figure 5:**
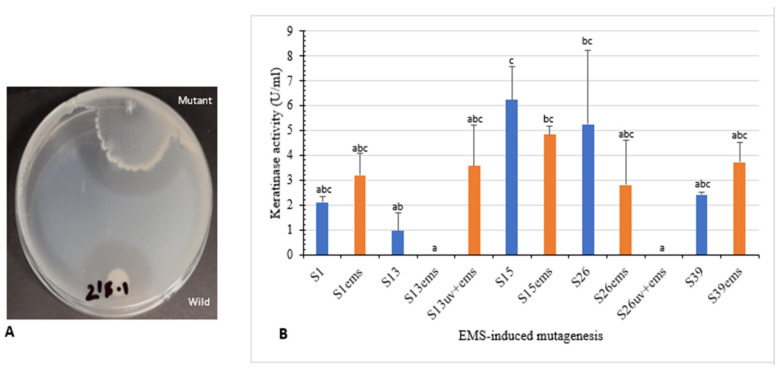
Effect of EMS-random mutagenesis on keratinase activity. (**A**) Wild and EMS-mutated isolate S39 showing clear zone of hydrolysis on skim milk agar. (**B**) Keratinolytic activity of wild, EMS and UV+EMS-mutated isolates. Bars are the standard error of the mean. Mean with the different letters are significantly different according to Duncan’s at *p* < 0.05.

Moreover, isolates S1, S13, S15, S26, and S39, as well as UV mutants (S13uv and S26uv) with higher keratinase activity than the wild type were treated with EMS. Clear zone hydrolysis using skim milk for the wild and mutated isolates is shown in Figure 5A. Keratinase activity was increased with the mutants S1ems (3.2 U/mL) S13uv+ems (3.5 U/mL), and S39ems (3.7 U/mL) compared with the wild isolates after 72 h of incubation (Figure 5B). Results of our study indicated that mutations by EMS increased keratinase activity from 1.5–3.7-fold compared with the wild isolates. The activity of keratinase after mutation by EMS from microbial origin was detected to be 1.4–1.9-fold activity of the wild type [42,43].

### 3.5. Evaluation of Biodegradation Efficiency of the Wild and Mutant Isolates by Digital Camera and Scanning Electron Microscopy (SEM)

**Figure 6:**
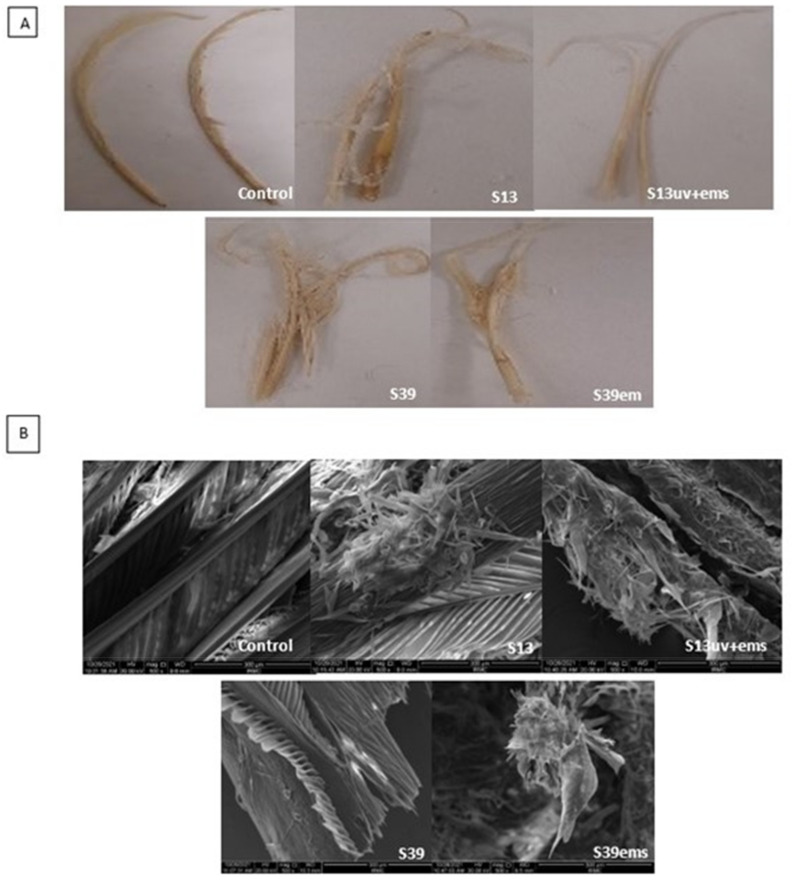
Evaluation of biodegradation efficiency of the wild and mutant isolates. (**A**) Feathers degradation after 72 h of incubation with isolates S13 and S39 and their mutants S13uv+ems and S39ems examined by digital camera and (**B**) scanning electron microscopy.

The digital photos and SEM micrographs showed a degradative action of S13, S13uv+ems, S39, and S39ems on the chicken feather as keratinous substrate (Figure 6). Data presented in Table 1 showed improvement in keratinase activity and feather degradation with the mutants S13uv+ems (4.0 U/mL and 65%) and S39ems (3.5 U/mL and 57.5%) compared with their wild isolates S13 (2.7 U/mL and 45%), and S39 (2.7 U/mL and 22.5%) after 72 h of incubation. Figure 6 depicts the deterioration of feather structure as a result of the keratinase activity. The rachis surface was disrupted and the advanced fragmentation and detachment of feather barbs were observed. However, as fermentation progressed, the barbules were partially degraded from the vane. Similarly, Bach et al. [44] reported that many strains did not achieve complete feather degradation as the rachis was not fully degraded. In contrast to this study, complete feather degradation was achieved by *Bacillus* sp. FPF-1 as the barbules and the vane were completely degraded within 72 h of fermentation [26]. Gupta and Singh [25] found that the keratinase of *B. subtilis* RSE165 hydrolyzed the hydrogen bonds of the outer and inner part of the quill and promotes the complete degradation of the feather after 72 h of incubation. The significance of the SEM imaging process includes proving the ability of our keratinolytic bacteria to hydrolyze the feather keratin and to understand the degradation pattern.

### 3.6. 16S rRNA Identification of the Keratinolytic Bacterial Isolates

The five keratinolytic bacterial isolates were identified through amplification and sequencing of the *16S rRNA* gene. Sequences were compared with those of the GenBank database using BLASTn; the 5 sequences were 100% identical and shared 99.82–100% identity to the species of *Bacillus cereus* group. In Saudi Arabia, Alshehri et al. [45] reported that *Bacillus* sp. BAM3 have been identified based on *16S rDNA* as *Bacillus cereus* BAM3 sharing 98.9–100% identity.

**Figure 7:**
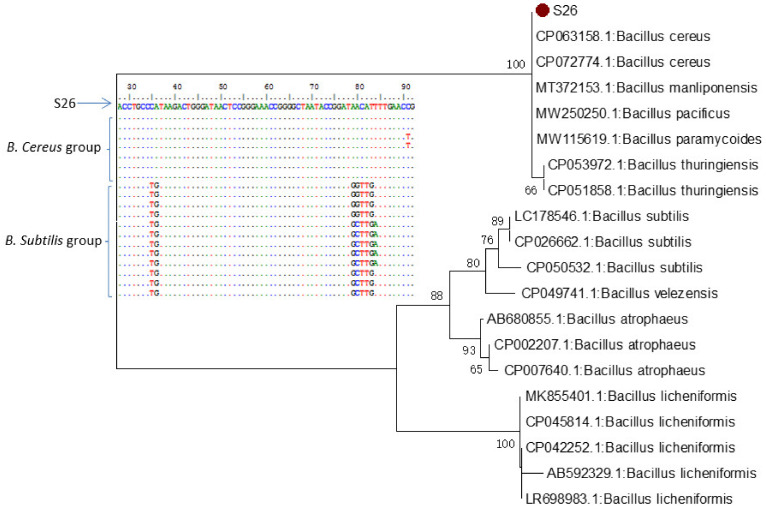
Multiple sequence and neighbor-joining phylogenetic analysis of *16S rRNA* gene strain S26 against sequences retrieved from GenBank database.

In addition, multiple sequence alignment and phylogenetic analysis against the *16S rRNA* sequence of *B. subtilis* and *B. cereus* groups retrieved from the GenBank database clearly showed the close relationship between our strains and those of the *B. cereus* group (Figure 7). Furthermore, 58 major substitution positions were detected and differentiated between *B. subtilis* and *B. cereus* groups in a region of 1209 nt (Supplementary Materials Figure S3). These positions could be used as markers to differentiate between species belonging to the *B. subtilis* group from those of the *B. cereus* group [46].

### 3.7. In Silico Characterization of the Wild-Type Keratinase KerS and Mutants

Keratinase (*KerS*) gene from strains S1, S13, S15, S26, and S39, as well as their corresponding mutants, was amplified and sequenced. Different bioinformatics tools were used to analyze various aspects of the *KerS* gene, which included sequence similarity, phylogenetic analysis, functional analysis, physicochemical characterization, and prediction of keratinase 3D structure and molecular docking.

#### 3.7.1. Keratinase Gene Similarity Search

Based on the similarity between our keratinase *KerS* gene sequences (98–100%), *KerS*13, *KerS*13uv+ems and *KerS*26uv were selected as representative sequences for further analysis. *KerS* gene is composed of 704–1194 nt and 234–397 aa. Using BLASTp, amino acid sequence analysis of *KerS* gene revealed a high level of identity (97.98–100%) with S8 family peptidase of *B. cereus* group (Supplementary Materials Table S1). S8 family peptidase is a subtilisin-like serine protease with a catalytic triad of Asp/His/Ser [47]. Gurunathan et al. [48] in silico identified keratin degrading subtilisin like serine alkaline protease from *B. cereus*. Fellahi et al. [10] found that the results of amino acid sequences alignment with known *B. pumilus* proteases indicated that the two keratinases *Ker*1 and *Ker*2 of *B. pumilus* strain C4 are subtilisin-like serine proteases belonging to the protease S8 family.

Pairwise alignment comparison between *KerS* gene (*KerS*13, *KerS*13uv+ems, and *KerS*26uv) and two keratinases retrieved from UniprotKB database showed identity between 97.59–99.10%. *KerS*26uv shared the highest identity (99.10% and 98.80% with keratinase *B. thuringiensis* (A0A1L6PVT6) and keratinase *ker* 6 *B. cereus* (F8SVT0), respectively).

#### 3.7.2. Multiple Sequence Alignment and Phylogenetic Analysis

**Figure 8:**
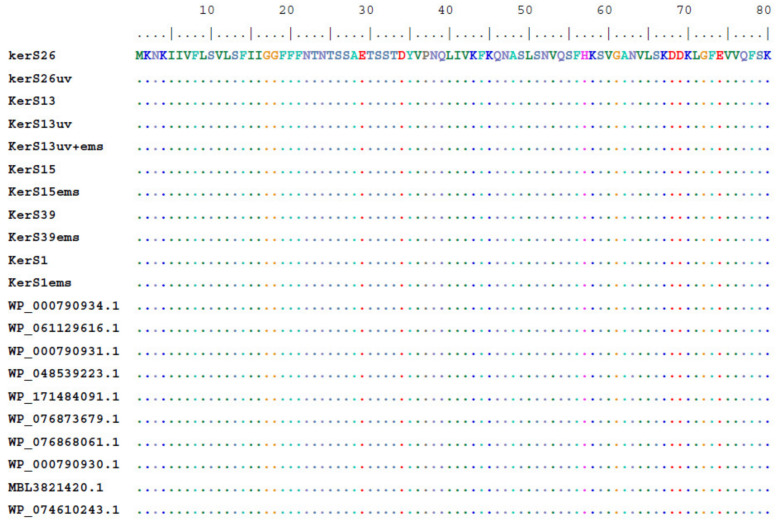

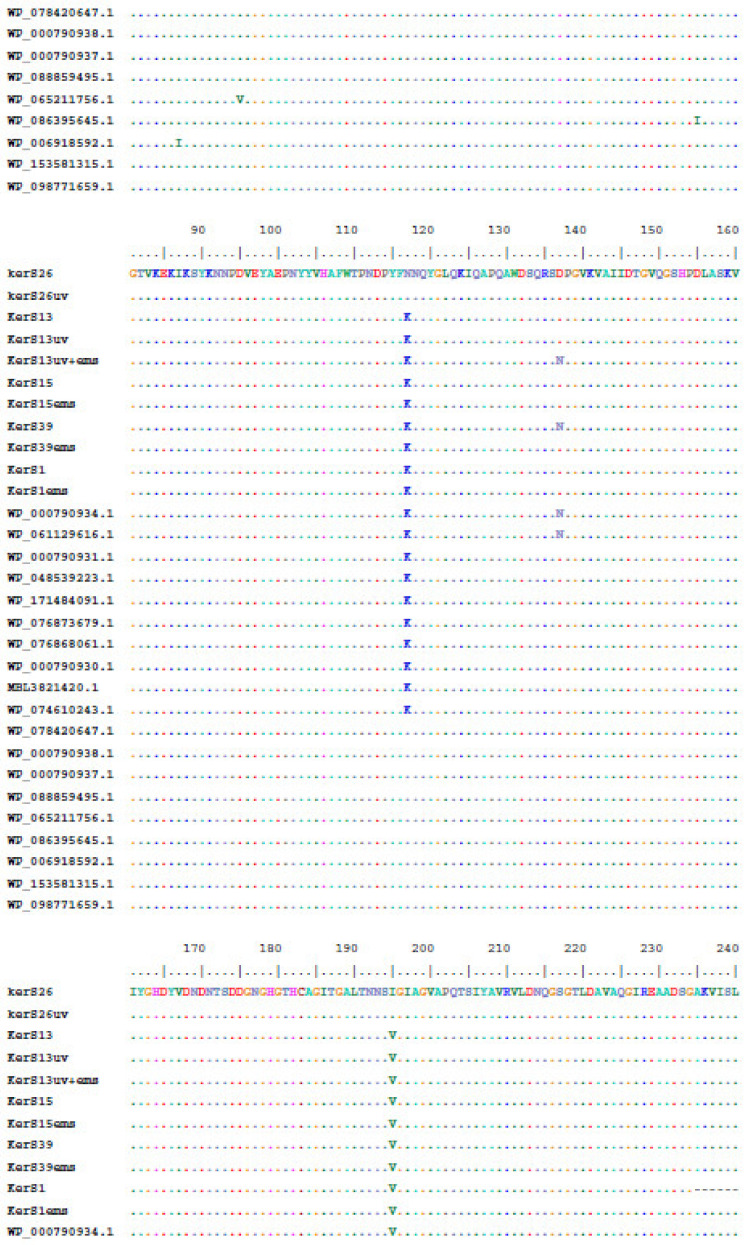

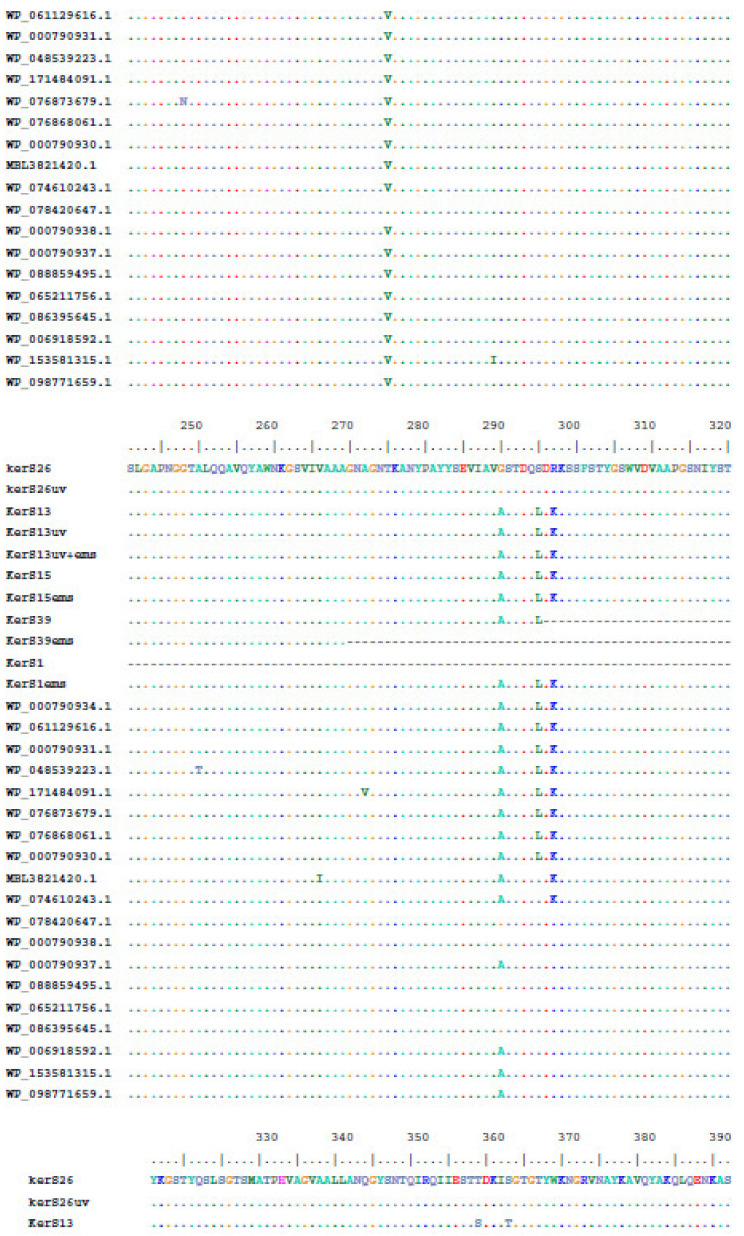

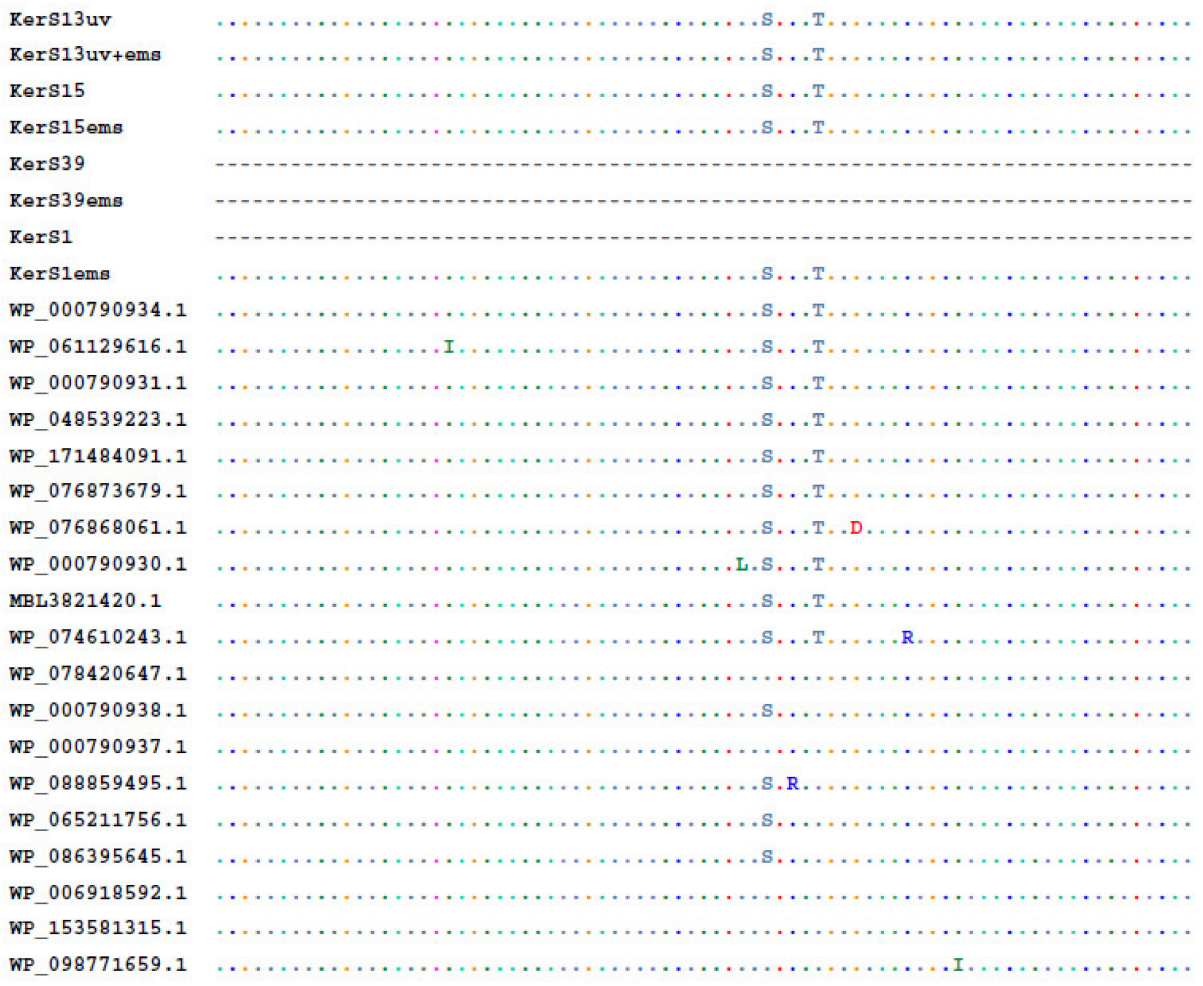
Multiple amino acid sequence analysis of *KerS* gene against S8 family peptidase *Bacillus cereus* group sequences retrieved from GenBank database.

**Figure 9:**
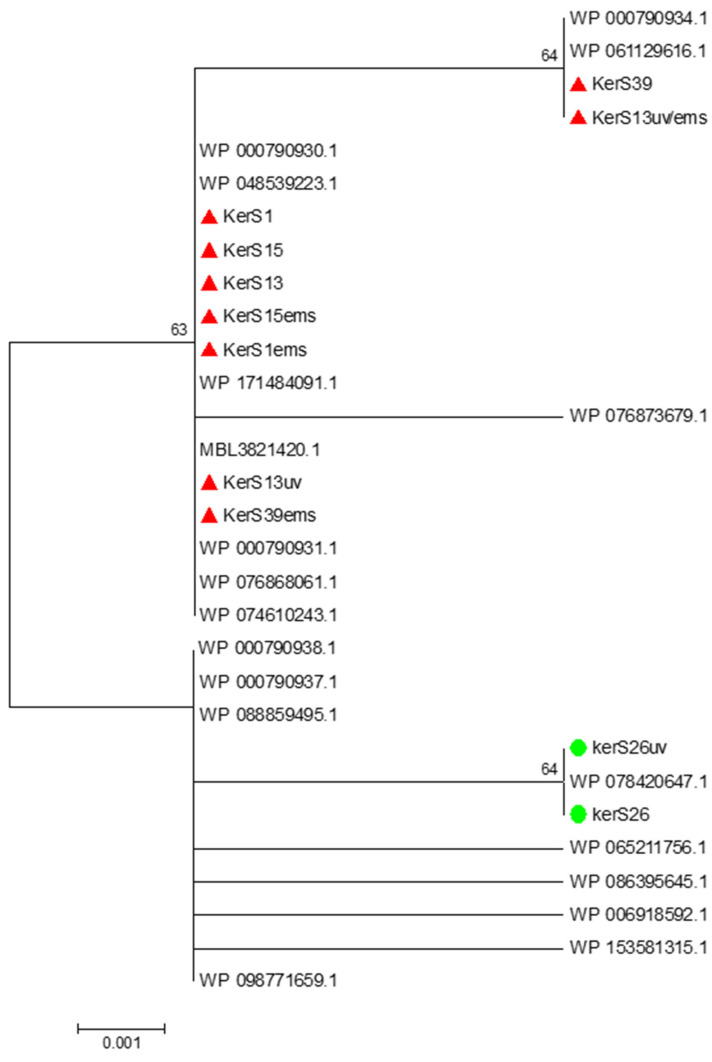
Neighbor-joining phylogenetic analysis of *KerS* gene against S8 family peptidase *Bacillus cereus* group sequences retrieved from GenBank database.

Multiple sequence alignment and phylogenetic analysis of our *KerS* gene and those of S8 family peptidase, *B. cereus* group, obtained from GenBank database are shown in Figure 8 and Figure 9. Comparison of *KerS* gene sequence of the wild and mutant strains showed D_137_N substitution in the mutant *KerS*13uv+ems but not in the wild *KerS*13. After mutagenesis, the activity of keratinase *KerS*13uv+ems was found to be a 3.73-fold activity of the wild type. Surprisingly, D_137_N substitution was detected in *KerS*39, serine protease WP_000790934.1, and WP_061129616.1 (Figure 8). Abdel-Naby et al. [32] demonstrated that combination of chemical/physical mutagenesis on *B. cereus* resulted in 19 amino acid substitutions that led to an improvement of the protease by about 31.17% compared with the wild type; nine of the amino acid substitutions include I_242_Y, K_244_R, D_245_A, K_246_R, G_248_N, R_253_V, T_260_H, W_279_R, and E_281_L and improved the catalytic efficiency of the enzyme. Furthermore, the introduction of amino acid substitutions by site-directed mutagenesis on the keratinase of *Stenotrophomonas maltophilia* (A218S and A218G), *B. licheniformis* (N122Y, N217S, A193P, and N160C), and *Brevibacillus parabrevis* (T218S, S236C, and N181D) exhibited high improvement in thermostability, enzyme production, and catalytic activity [49,50,51].

Moreover, seven substitutions (N_117_K, V_195_I, A_290_G, S_295_L, R_297_K, T_364_S, and S_368_T) observed in the study distinguished *KerS*26 and its mutant *KerS*26uv from other keratinase sequences and serine proteases, S8 family peptidase (Figure 8). The seven substitutions were also detected in the serine proteases WP_000790937.1, WP_088859495.1, WP_065211756.1, WP_086395645.1, WP_006918592.1, WP_153581315.1, and WP_098771659.1 (Figure 8). Accordingly, keratinase *KerS* gene sequences were separated into two groups (Figure 9). The diversity between our keratinase sequences was 0.025, however, it was 0.003–0.022 between our keratinase and those of the serine protease, S8 family peptidase *B. cereus* group (Supplementary Materials Figure S4). Although the mutant S26uv had 1.73-fold more keratinase activity than the wild S26 strain, no substitutions were detected in keratinase *KerS*26uv compared to *KerS*26.

#### 3.7.3. Functional Analysis of Keratinase *KerS* Gene

**Figure 10:**
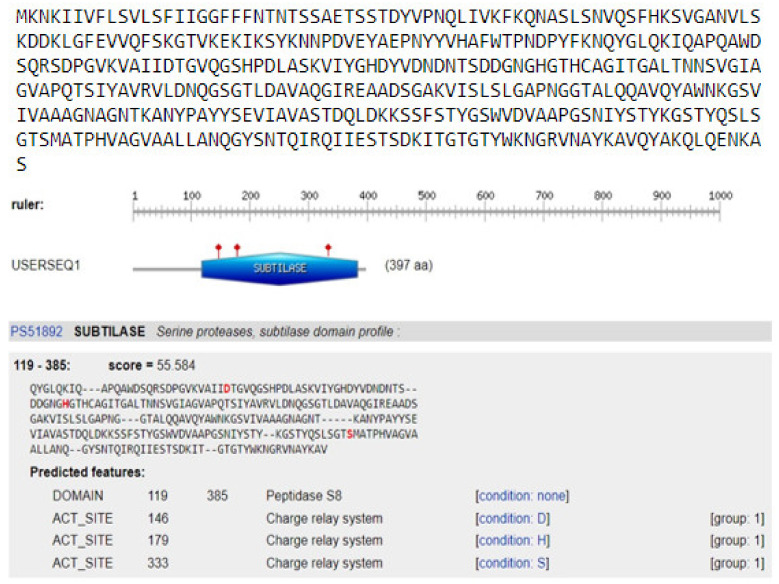
Prediction of the catalytic domain and active sites of keratinase gene *KerS*13. The catalytic triad subtilase ASP146, subtilase HIS179 and subtilase SER333 with a score of 55.5.

In order to identify protein domains, families, and functional sites, the ScanProsite search tool was used to scan *KerS* gene for matches against PROSITE profiles and patterns. Functional prediction of keratinase gene resulted in the detection of serine protease subtilase domain (peptidase S8) at amino acid position 119–385 of *KerS* gene, including the catalytic triad subtilase ASP146, subtilase HIS179, and subtilase SER333 (Figure 10). Most of the keratinases are found in the subfamily S8A including 14 keratinases; their active site contains the catalytic triad of Asp, His, and Ser [33]. The catalytic triad plays an essential role in the catalytic mechanism. The triad is positioned in the active site of the enzyme where catalysis takes place and is conserved in all superfamilies of serine protease enzymes [52]. Interestingly, the detected substitutions in *KerS* gene (Figure 8) did not affect the prediction of the subtilase domain and the catalytic triad, and accordingly, we suggest that these substitutions did not affect the *KerS* function. According to the close relationship between *KerS* and peptidase S8 *B. cereus* group and the prediction of serine protease subtilase family domain and catalytic tried, we suggest that the *KerS* gene belongs to serine protease S8 family peptidase. Keratinases belong to the serine- and metalloprotease families. The major keratinase family is the S8 family, and the subtilisin subfamily members, in particular, have keratinolytic activity [7].

**Figure 11:**
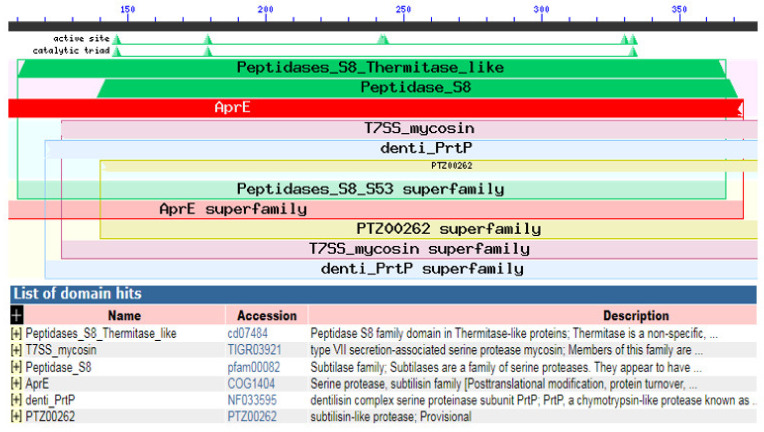
NCBI conserved domains search analysis showing the similarity between our *KerS* gene and peptidases S8 thermitase-like.

Using InterProScan and NCBI conserved domains search, our *KerS* showed similarity to peptidases S8 thermitase-like (thermitase-like domain) at position 110–368 (Figure 11). Thermitase is a serine protease belonging to family S8 (subtilases) [53], which includes subtilisin isolated from *Bacillus* species. Thermitase is a thermostable serine proteinase that is more stable against thermal denaturation and proteolytic degradation than most members of the family [54]. Furthermore, pairwise alignment comparison revealed a high identity between *KerS* gene and two thermostable serine proteases; thermophilic serine proteinase *B. cereus* (B3ZJ21) and thermitase alkaline serine protease *B. cereus* retrieved from the UniprotKB database (95.58% and 99.09%, respectively). Interestingly, the mutant *KerS*13uv+ems shared the highest identity with thermitase alkaline serine protease (100%). However, thermophilic serine proteinase B3ZJ21 showed the highest identity with *KerS*13 (95.78%). *KerS*26uv shared 95.48% and 97.59% identity with thermophilic and thermitase protease, respectively. According to Li et al. [47], the keratinase gene (*kerT*1) shared over 70% identity with the peptidase S8 thermitase family domain and possibly belongs to the serine endoprotease.

#### 3.7.4. Physicochemical Characterization of Keratinase *KerS* Gene

S8 family peptidase protein sequences representing different *B. cereus* group species were retrieved from NCBI-BLASTp and compared with the keratinase *KerS* gene to better investigate *KerS* gene encoding *KerS*13, *KerS*13uv+ems, and *KerS*26uv (Supplementary Materials Table S2). The physicochemical parameters were generated using ProtParam tools of the Expasy server to determine the number of amino acids, molecular weight, the total number of negatively and positively charged amino acids, theoretical *pI*, grand average of hydropathicity (Gravy), instability, and finally, the aliphatic index (Table 2).

*KerS* gene and the other peptidase S8 serine proteases composed of 397aa with molecular weight ranged from 42.2–42.4 kDa. The molecular weight of the keratinase gene ranges from 18–200 kDa [55]. *Bacillus* species are of medium sizes, such as 33 kDa (*B. licheniformis*) and 39 kDa (*B. thuringiensis*) [56,57]. According to Banerjee et al. [58], negatively and positively charged amino acids determine the structural diversity. The number of positively charged amino acids (Arg + Lys) was higher than the negatively charged amino acids (Asp + Glu). Banerjee et al. [58] found that positively charged amino acids of *B. licheniformis*, *B. pumilus*, and *B. mojavensis* were higher than negatively charged residues. The range of theoretical *pI* was identified between 7.05 and 8.57; *KerS*13uv+ems showed the highest *pI* (8.57) compared with *KerS*13 (8.28) and *KerS*26uv (7.72); thus, the calculated *pI* indicates that keratinase *KerS* possesses an alkaline character with a correlation to the pH stability at 8–9. GRAVY index showed a low-value range from −0.32 to −0.35. A low GRAVY index indicates low hydrophobicity and high hydrophilicity of the protein, suggesting a better interaction with water. The predicted instability index < 40 indicates that the protein is stable, whereas values > 40 suggest that the protein is unstable [59]. All the protein sequences showed an instability index value of less than 40 (17.34–20.60) suggesting protein stability. *KerS*13uv+ems showed the lowest instability index (17.48) compared with *KerS*13 (17.56) and *KerS*26uv (19.28). The aliphatic index is measured by the relative volume occupied by the aliphatic side chains, which indicates the degree of thermal stability of a protein, and this is wholly dependent on the relative volume of a protein occupied by the aliphatic residues [60,61]. The predicted aliphatic index was 73.95–76.17 which indicates keratinase thermostability. Keratinase of *B. licheniformis* and *Bacillus* sp. showed an instability index of 12.61 and 22.69 and aliphatic index of 83.69 and 60.53, respectively, which implied enzyme stability and significant thermostability [58,62]. The physiochemical characteristics such as the instability, gravy, and aliphatic index, as well as the similarity to thermitase domain, thermitase, and thermophilic serine proteases, revealed the stability and significant thermostability of our *KerS* enzyme. Interestingly, *KerS*uv+ems shared the highest identity (100%) to the thermitase gene, the lowest instability, and the highest aliphatic index reflecting the high thermostability of this mutant which may be enhanced by the uv+ems mutagenesis. It was reported that the introduction of amino acid substitutions by site-directed mutagenesis on the keratinase gene exhibited high improvement in thermostability, enzyme production, and catalytic activity [49,50,51].

#### 3.7.5. Structure Modeling and Analysis of Wild-Type Keratinase and Mutants

**Figure 12:**
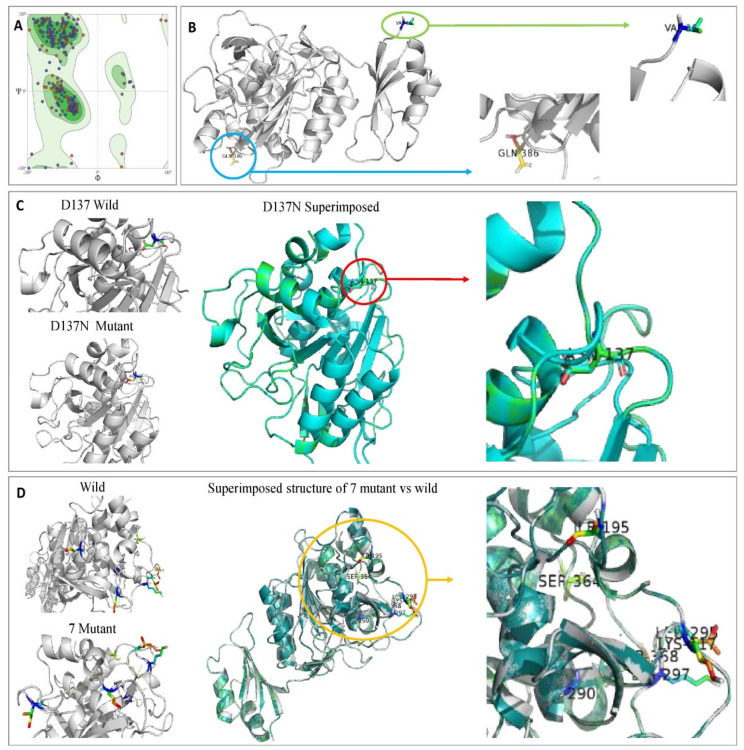
(**A**) Model validation of keratinase 3D structure by Ramachandran plot showing 91.98% favored region and 1.43% of the disallowed region. Amino acids not in the favored region are A324 SER, A46 GLN, A160 VAL, A137 ASP, and A114 PRO. (**B**) Ribbon diagrams of the modeled keratinase showing α-Helices, β-strands, and loop. (**C**) Superimposed structure of *KerS*13uv+ems (D_137_N). (**D**) Superimposed structure of seven mutants that differentiate between *KerS*26uv and the other keratinase strains.

Ramachandran plot was conducted to validate the stereochemical stability of keratinase structure and to explain the structure of the keratinase of the wild and mutant strains. Figure 12A showed 91.98% of the amino acid residue in the most favored region, and 1.43% of the remaining residues in the outlier region, which indicates the model is of good quality and stability for in silico studies. The quality factor of the predicted model was observed as 83.09 using ERRAT2. Gupta et al. [63] validated the modeled structure of the *B. subtilis* RSE163 keratinase gene using Ramachandran’s plot and results showed that 305 of the amino acid residues (84.3%) were in the favored region. Compared with the previous study by Gupta et al. [63], the current model is relatively better as 91.98% of the amino acid residue is in the most favored region.

#### 3.7.6. Molecular Docking Study of Keratinase *KerS* Gene

**Figure 13:**
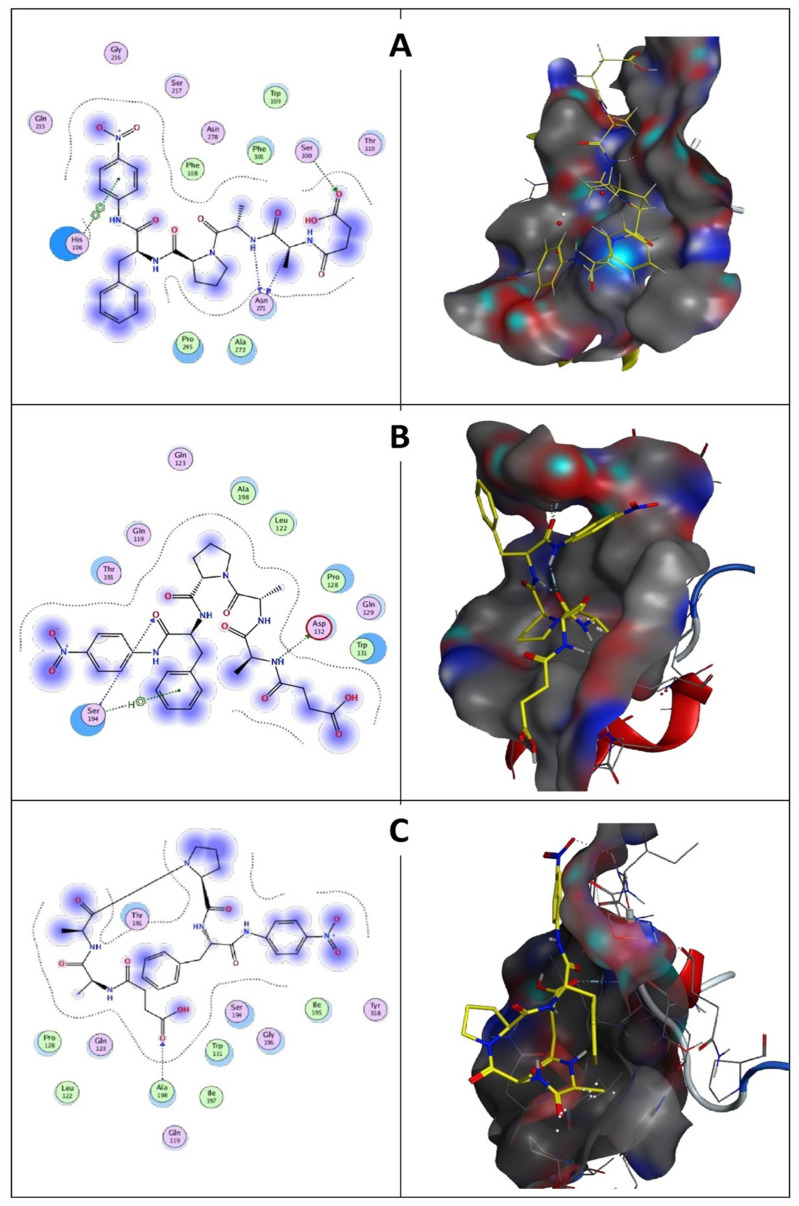
(**A**) Wild keratinase ligand interaction: left is the 2D structure and right is the 3D structure. Ligand has three hydrogen bonds: one with Ser 300 and two with Asn 271. (**B**) D_137_N keratinase ligand interaction: left is the 2D structure and right is the 3D structure. Ligand has two hydrogen bonds: Asp 132 and Ser 194. (**C**) The 7 mutants keratinase ligand interaction: left is the 2D structure and right is the 3D structure. Ligand has one hydrogen bond with Ala 190.

Molecular docking results indicate that no mutations occurred in the active sites (GLN39 LEU65 SER66 LYS67 SER79 ASN102 TYR104 ASP165 TYR166 VAL167 ASP168 ASN169 ASP170 VAL211 ASP213 ASN214 SER217 GLY218 THR219 ASP221 ALA222 GLN225) of the predicted wild keratinase compared with the mutants (Figure 12B). The RMSD values and the superimposed structures (Figure 12C,D) indicate that the mutant proteins D_137_N (0.003 Å) and the seven mutants (0.006 Å) are structurally slightly different from the wild keratinase. However, the mutant of *KerS*13uv+ems (D_137_N) is not structurally much different from the seven mutants of *KerS*26uv as the substitutions did not affect the functional domains of the keratinase active site (Figure 12C,D). Data presented in Table 3 showed a slight increase in the binding affinity of the mutant proteins D_137_N of *KerS*13uv+ems and the seven mutants of the *KerS*26uv as they exhibited an affinity score of (−7.17, −7.43), respectively, compared with the wild protein (−6.57). Moreover, the E score2 values of the mutant proteins indicate that they are a minor increase in the binding compared with the wild proteins. Furthermore, Figure 13 showed the interaction between the keratinase protein and the ligand, the position of the residues (Ser 300, Asn 271) of wild keratinase, (Asp 132, Ser 194) of D_137_N, and (Ala 190) of seven mutants that showed the hydrogen bond with the substrate. The docking results of *Bacillus* sp. showed involvement of Asp160 and Try246 amino acid residues in the binding with all compounds and involvement of the other important residues such as His85, Asp97, Glu131, Asp160, Glu132, Tyr246, and His247 in hydrogen bonding with the substrates [64]. Moreover, the docking results of mutated *Stenotrophomonas maltophilia KerS*MD revealed that positions of Y94, Y187, and Y215 had direct interaction with the propeptide since it is possible that appropriate binding pockets can enhance the maturation and folding rate of keratinase [49].

## 4. Conclusions

In conclusion, novel feather degrading keratinase isolated from different keratinolytic bacteria was characterized in vitro and in silico. Physical and chemical mutagenesis resulted in efficient mutants with high keratinase activity and remarkable feather hydrolysis compared with the wild type. Sequence analysis demonstrated that the keratinase *KerS* gene is a serine protease peptidase S8 family of *B. cereus* group with subtilase domain and a typical catalytic triad (Asp, His, and Ser). D_137_N substitution was observed in the *kerS* gene *KerS*13uv+ems, as well as seven different substitutions in *KerS*26 and its mutant *KerS*26uv compared with the other *KerS* gene sequences; the predicted substitutions did not affect the subtilase domain and the active site of the keratinase gene. The predicted low instability index, high aliphatic index, and low GRAVY value of the *KerS* gene, as well as similarity to thermostable proteases imply that this enzyme is highly thermostable and has excellent solubility in water. Docking analysis confirmed the binding affinity of keratinase *KerS*13uv+ems and *KerS*26uv and substrate. Therefore, the new keratinases *KerS*13uv+ems and *KerS*26uv presented high keratinolytic activity, efficiency in feather degradation, thermostability and binding affinity providing biotechnological potential as an effective and environmentally friendly alternative to the conventional chemicals used in keratin hydrolysis.

## Figures and Tables

**Table 1:** Evaluation of feather degrading ability of S13uv+ems and S39ems and their wild type for SEM analysis.

Bacterial Strains	pH	Feather Hydrolysis (%)	Keratinase Activity (U/mL)
S13	9.55 ± 0.02 ^ab^	45.00 ± 0.00 ^b^	2.72 ± 0.20 ^a^
S13uv+ems	9.59 ± 0.07 ^ab^	65.00 ± 0.00 ^d^	4.07 ± 0.71 ^a^
S39	9.18 ± 0.11 ^a^	22.50 ± 2.50 ^a^	2.71 ± 0.62 ^a^
S39ems	9.69 ± 0.18 ^b^	57.50 ± 2.50 ^c^	3.53 ± 0.18 ^a^
*p*-value	0.101	0.000	0.138

Values are expressed as means ± standard error. Mean with the different letters are significantly different according to Duncan’s at *p* < 0.05.

**Table 2:** Physiochemical properties of keratinase *KerS* primary structure compared with the similar serine proteases of *B. cereus* group retrieved from GenBank database.

Serine Protease		N Amino Acids	Molecular Weight	Asp + Glu/Arg + Lys	Theoretical pI	Gravy Index	Instability Index	Aliphatic Index
*KerS*13 *B. cereus* group		397	42,345.95	30/32	8.28	−0.32	17.56	75.69
1	*B. cereus group*	397	42,344.96	29/32	8.57	−0.32	17.94	75.69
2	*B. paranthracis*	397	42,374.00	30/32	8.28	−0.32	17.34	76.17
3	*B. thuringiensis*	397	42,275.77	30/31	7.72	−0.33	17.94	74.96
4	*B. anthracis*	397	42,345.95	30/32	8.28	−0.32	17.98	75.94
5	*B. tropicus*	397	42,331.92	30/32	8.28	−0.32	20.60	75.94
6	*B. cereus*	397	42,333.89	30/32	8.28	−0.34	19.09	74.96
*KerS*13uv+ems *B. cereus* group		397	42,344.96	29/32	8.57	−0.32	17.48	75.69
1	*B. cereus*	397	42,358.99	29/32	8.57	−0.32	18.59	75.94
2	*B. thuringiensis*	397	42,333.81	30/31	7.72	−0.34	18.90	74.71
3	*B. paranthracis*	397	42,348.86	31/32	7.72	−0.34	20.39	74.71
4	*B. cereus group*	397	42,319.78	30/31	7.72	−0.34	19.28	74.46
5	*B. cereus*	397	42,333.89	30/32	8.28	−0.34	18.88	74.96
6	*B. anthracis*	397	42,390.94	30/32	8.28	−0.35	17.80	74.71
*KerS*26uv *B. cereus* group		397	42,333.81	30/31	7.72	−0.34	19.28	74.71
1	*B. cereus*	397	42,333.81	30/31	7.72	−0.34	19.28	74.71
2	*B. bombysepticus*	397	42,305.75	30/30	7.05	−0.33	19.76	74.71
3	*B. thuringiensis*	397	42,365.81	30/31	7.72	−0.35	18.85	73.95
4	*B. paranthracis*	397	42,319.87	30/32	8.28	−0.34	18.88	74.71
5	*B. toyonensis*	397	42,261.74	30/31	7.72	−0.33	19.26	74.96
6	*B. fungorum*	397	42,289.84	30/32	8.28	−0.33	19.36	74.96
7	*B. tropicus*	397	42,390.94	30/32	8.28	−0.35	18.18	74.71
8	*B. toyonensis*	397	42,422.95	30/32	8.29	−0.35	19.60	73.98

**Table 3:** The molecular docking of *N*-succinyl-l-alanyl-l-alanyl-l-prolyl-l phenylalanine 4 nitroanilide S-9205 against keratinase.

	Logp	Logs	Affinity	S (kcal/mol)	RMSD_Refine	E_Conf	E_Place	E_Score1	E_Refine	E_Score2
Wild	1.13	−5.76	−6.57	−6.6864	1.7138	−16.7596	−27.6505	−7.5627	−39.8656	−6.6864
D137N	1.13	−5.76	−7.17	−6.5408	1.5868	−22.9948	−58.3634	−8.0156	−36.007	−6.5408
7 Mutant	1.13	−5.76	−7.43	−7.17	7.0603	−17.7788	−56.0579	−5.0721	−41.3177	−7.17

Logp—the log octanol/water partition coefficient; Logs—Log of the aqueous solubility (mol/L); S—the final score of GBVI/WSA binding free energy; RMSD_Refine—the mean square deviation after refinement; E_Conf—energy conformer; E_Place—score of the placement phase; E_Score1—score of the first step of notation; E_Refine—score of refinement; and E_score2—score of the second step of notation.

## Data Availability

Not applicable.

## Supplementary Materials

The following are available online at https://www.mdpi.com/article/10.3390/microorganisms10010093/s1. Figure S1: Effect of Glucose, NH_4_CL, and MgSO_4_·7H_2_O individually and in combination on keratinase activity by isolates S1, S15, and S26. Figure S2: Influence of different keratin substrate (white chicken feather, black chicken feather, white sheep wool, black sheep wool, and human hair) on keratinase production and chicken feather degradation after 72 h incubation at 45 °C (A) Degradation of a white feather, (B) control, (C) degradation of black chicken feather, (D) control, (E) degradation of white sheep, (F) control, (G) degradation of black sheep wool, (H) control, (I) degradation of human hair and (J) control. Figure S3: Multiple sequence analysis of *16S rRNA* gene, strain S26 against sequences retrieved from GenBank database. Stars correspond to the 58 major substitution position differentiated between *B. subtilis* and *B. cereus* groups. Strain S26 is identical to *B. cereus* groups sequences. Figure S4: Estimates of Evolutionary Divergence of *KerS* gene against S8 family peptidase, *Bacillus cereus* group sequences retrieved from GenBank database. Table S1: BLASTp analysis of keratinase *kerS* showing deduced amino acid, total score, query cover, E-value and percentage of identity. Table S2: BLASTp analysis of *KerS* gene (*KerS*13, *KerS*13uv+ems, and *KerS*26uv) and the corresponding similar peptidase S8 *B. cereus* group species.

## References

[1] Tamreihao K., Mukherjee S., Khunjamayum R., Devi L.J., Asem R.S., Ningthoujam D.S. (2019). Feather degradation by keratinolytic bacteria and biofertilizing potential for sustainable agricultural production. J. Basic Microbiol..

[2] Kalaikumari S.S., Vennila T., Monika V., Chandraraj K., Gunasekaran P., Rajendhran J. (2019). Bioutilization of poultry feather for keratinase production and its application in leather industry. J. Clean. Prod..

[3] Navone L., Speight R. (2018). Understanding the dynamics of keratin weakening and hydrolysis by proteases. PLoS ONE.

[4] Tseng F.C. (2011). Biofibre Production from Chicken Feather. Master’s Thesis.

[5] Mamangkey J., Suryanto D., Munir E., Mustopa A.Z. (2019). Isolation, Molecular Identification and Verification of Gene Encoding Bacterial Keratinase from Crocodile (*Crocodylus porosus*) Feces. Proceedings of the 4th International Conference on Biological Sciences and Biotechnology, Medan, Indonesia, 8–9 December 2018.

[6] Lange L., Huang Y., Busk P.K. (2016). Microbial decomposition of keratin in nature—A new hypothesis of industrial relevance. Appl. Microbiol. Biotechnol..

[7] Qiu J., Wilkens C., Barrett K., Meyer A.S. (2020). Microbial enzymes catalyzing keratin degradation: Classification, structure, function. Biotechnol. Adv..

[8] Jaouadi N.Z., Rekik H., Badis A., Trabelsi S., Belhoul M., Yahiaoui A.B., Aicha H.B., Toumi A., Bejar S., Jaouadi B. (2013). Biochemical and molecular characterization of a serine keratinase from *Brevibacillus Brevis* US575 with promising keratin-biodegradation and hide-dehairing activities. PLoS ONE.

[9] Tang Y., Guo L., Zhao M., Gui Y., Han J., Lu W., Dai Q., Jiang S., Lin M., Zhou Z. (2021). A novel thermostable keratinase from Deinococcus geothermalis with potential application in feather degradation. Appl. Sci..

[10] Fellahi S., Chibani A., Feuk-Lagerstedt E., Taherzadeh M.J. (2016). Identification of two new keratinolytic proteases from a *Bacillus pumilus* strain using protein analysis and gene sequencing. AMB Express.

[11] Ahmadpour F., Yakhchali B., Musavi M.S. (2016). Isolation and identification of a keratinolytic *Bacillus cereus* and optimization of keratinase production. J. Appl. Biotechnol. Rep..

[12] Sutoyo S., Subandi S., Ardyati T., Suharjono S. (2019). Isolation and identification of keratinolytic bacteria from Jember, Indonesia as a biodegradation agent of chicken feather wastes. Asian J. Agric. Biol..

[13] Abdel-Fattah A.M., El-Gamal M.S., Ismail S.A., Emran M.A., Hashem A.M. (2018). Biodegradation of feather waste by keratinase produced from newly isolated *Bacillus licheniformis* ALW1. J. Genet. Eng. Biotechnol..

[14] De Paiva D.P., de Oliveira S.S., Mazotto A.M., Vermelho A.B., de Oliveira S.S. (2019). Keratinolytic activity of *Bacillus subtilis* LFB-FIOCRUZ 1266 enhanced by whole-cell mutagenesis. 3 Biotech.

[15] Kothari D., Rani A., Goyal A., Pandey A., Negi S., Soccol C. (2017). Keratinases. Current Developments in Biotechnology and Bioengineering, Production, Isolation and Purification of Industrial Products.

[16] Isiaka A., Adelere A. (2016). Keratinases: Emerging trends in production and applications as novel multifunctional biocatalysts. Kuwait J. Sci..

[17] Subugade S., Gupta S.G., Mokashe S. (2017). Isolation and Screening of Keratinase Producing Bacteria from Chicken Feather Dumping Site. Int. J. ChemTech Res..

[18] Reyes A., Ambita I.D., Batalon J.L., Aba B.L., Cortes A., Macabecha C.G., Montecillo A. (2018). Isolation and characterization of keratinolytic bacteria from soil samples of poultry waste dumping sites. Int. J. Agric. Technol..

[19] Rajak R.C., Malviya H.K., Deshpande H., Hasija S.K. (1992). Keratinolysis by *Absidia cylindrospora* and *Rhizomucor pusillus*: Biochemical proof. Mycopathologia.

[20] Aly M.M., Khalel A., Hassan S.M. (2019). Isolation, identification, and characterization of a keratolytic bacterium from poultry wastes. IOSR J. Pharm. Biol. Sci..

[21] Preczeski K.P., Dalastra C., Czapela F.F., Kubeneck S., Scapini T., Camargo A.F., Zanivan J., Bonatto C., Stefanski F.S., Venturin B. (2020). *Fusarium oxysporum* and *Aspergillus* Sp. as keratinase producers using swine hair from agroindustrial residues. Front. Bioeng. Biotechnol..

[22] Dhiva S., Ranjith K.R., Prajisya P., Sona K.P., Narendrakumar G., Prakash P., Emilin Renitta R., Samrot A.V. (2020). Optimization of keratinase production using *Pseudomonas aeruginosa* Su-1 having feather as substrate. Biointerface Res. Appl. Chem..

[23] Dagnaw M., Andualem B. (2019). Solid state fermentation of keratinolytic protease production using *Bacillus* Spp. isolated from water of leather processing ponds in North Gondar, Ethiopia. Biotechnol. Int..

[24] Aly M.M., Tork S. (2018). High Keratinase production and keratin degradation by a mutant strain KR II, derived from *Streptomyces radiopugnans* KR 12. Artic. J. Appl. Biol. Sci..

[25] Gupta S., Singh R. (2014). Hydrolyzing proficiency of keratinases in feather degradation. Indian J. Microbiol..

[26] Nnolim N.E., Okoh A.I., Nwodo U.U. (2020). *Bacillus* Sp. FPF-1 Produced keratinase with high potential for chicken feather degradation. Molecules.

[27] Gumilar J., Triatmojo S., Yusiati L.M., Pertiwiningrum A. (2015). Isolation, identification and dehairing activity of indonesian native keratinolytic bacteria *Exiguobacterium* Sp. DG1. Pak. J. Biotechnol..

[28] AlJindan R., AlEraky D.M., Borgio J.F., AbdulAzeez S., Abdalhamid B., Mahmoud N., Farhat M. (2021). Diagnostic Deficiencies of *C. difficile* Infection among Patients in a Tertiary Hospital in Saudi Arabia: A Laboratory-Based Case Series. Saudi J. Biol. Sci..

[29] Sigrist C.J., De Castro E., Cerutti L., Cuche B.A., Hulo N., Bridge A., Bougueleret L., Xenarios I. (2013). New and continuing developments at PROSITE. Nucleic Acids Res..

[30] Abdul Azeez S., Alhashim Z.G., Al Otaibi W.M., Alsuwat H.S., Ibrahim A.M., Almandil N.B., Borgio J.F. (2020). State-of-the-art tools to identify druggable protein ligand of SARS-CoV-2. Arch. Med. Sci..

[31] Saibabu V., Niyonzima F., More S. (2013). A laser-photolysis fragment-fluorescence (LPFF) method for the detection of gaseous nitric acid in ambient air. J. Atmos. Chem..

[32] Abdel-Naby M.A., El-Wafa W.M., Salim G.E. (2020). Molecular characterization, catalytic, kinetic and thermodynamic properties of protease produced by a mutant of *Bacillus cereus*-S6-3. Int. J. Biol. Macromol..

[33] Martinez J.P., Cai G., Nachtschatt M., Navone L., Zhang Z., Robins K., Speight R. (2020). Challenges and opportunities in identifying and characterising keratinases for value-added peptide production. Catalysts.

[34] Elkomy H., Al-Dosary S., El-Naghy M., Abdelhamid M., Immam M. (2019). Optimization of azo-keratin hydrolysis by alginate-immobilized keratinase produced from *Bacillus licheniforms*. J. Adv. Biomed. Pharm. Sci..

[35] Karray A., Alonazi M., Horchani H., Bacha A.B. (2021). A novel thermostable and alkaline protease produced from *Bacillus stearothermophilus* isolated from olive oil mill sols suitable to industrial biotechnology. Molecules.

[36] Mazotto A.M., Coelho R.R., Cedrola S.M., De Lima M.F., Couri S., De Souza E.P., Vermelho A.B. (2011). Keratinase production by three *Bacillus* spp. using feather meal and whole feather as substrate in a submerged fermentation. Enzyme Res..

[37] Suntornsuk W., Suntornsuk L. (2003). Feather degradation by *Bacillus* sp. FK 46 in submerged cultivation. Bioresour. Technol..

[38] El-Refai H., AbdelNaby M., Gaballa A., El-Araby M., Abdel Fattah A. (2005). Improvement of the newly isolated *Bacillus pumilus* FH9 keratinolytic activity. Process Biochem..

[39] Park G.T., Son H.J. (2009). Keratinolytic activity of *Bacillus megaterium* F7-1, a feather-degrading mesophilic bacterium. Microbiol. Res..

[40] Williams C.M., Richter C.S., Mackenzie J.M., Shih J.C. (1990). Isolation, identification, and characterization of a feather-degrading bacteriumt. Appl. Environ. Microbiol..

[41] Zeng Y.H., Shen F.T., Tan C.C., Huang C.C., Young C.C. (2011). The flexibility of UV-inducible mutation in *Deinococcus ficus* as evidenced by the existence of the ImuB-DnaE2 gene cassette and generation of superior feather degrading bacteria. Microbiol. Res..

[42] Afifi A.F., Abo-Elmagd H.I., Housseiny M.M. (2014). Improvement of alkaline protease production by *Penicillium chrysogenum* NRRL 792 through physical and chemical mutation, optimization, characterization and genetic variation between mutant and wild-type strains. Ann. Microbiol..

[43] Duarte T.R., Oliveira S.S., Macrae A., Cedrola S.M., Mazotto A.M., Souza E.P., Melo A.C., Vermelho A.B. (2011). Increased expression of keratinase and other peptidases by *Candida parapsilosis* mutants. Braz. J. Med. Biol. Res..

[44] Bach E., Cannavan F.S., Duarte F.R., Taffarel J.A., Tsai S.M., Brandelli A. (2011). Characterization of feather-degrading bacteria from Brazilian soils. Int. Biodeterior. Biodegrad..

[45] Alshehri W.A., Khalel A., Elbanna K., Ahmad I., Abulreesh H.H. (2021). Bio-plastic films production from feather waste degradation by keratinolytic bacteria *Bacillus cereus*. J. Pure Appl. Microbiol..

[46] Mahmoud A., Kotb E., Alqosaibi A.I., Al-Karmalawy A.A., Al-Dhuayan I.S., Alabkari H. (2021). In Vitro and in silico characterization of alkaline serine protease from *Bacillus subtilis* D9 recovered from Saudi Arabia. Heliyon.

[47] Li H.J., Tang B.L., Shao X., Liu B.X., Zheng X.Y., Han X.X., Li P.Y., Zhang X.Y., Song X.Y., Chen X.L. (2016). Characterization of a new S8 serine protease from marine *Sedimentary photobacterium* sp. A5-7 and the function of its protease-associated domain. Front. Microbiol..

[48] Gurunathan R., Huang B., Ponnusamy V.K., Hwang J.S., Dahms H.U. (2021). Novel recombinant keratin degrading subtilisin like serine alkaline protease from *Bacillus cereus* isolated from marine hydrothermal vent crabs. Sci. Rep..

[49] Fang Z., Zhang J., Du G., Chen J. (2017). Rational protein engineering approaches to further improve the keratinolytic activity and thermostability of engineered keratinase *KerS*MD. Biochem. Eng. J..

[50] Liu B., Zhang J., Fang Z., Gu L., Liao X., Du G., Chen J. (2013). Enhanced thermostability of keratinase by computational design and empirical mutation. J. Ind. Microbiol. Biotechnol..

[51] Zhang R.X., Gong J.S., Su C., Qin J., Li H., Li H., Shi J.S., Xu Z.H. (2020). Recombinant expression and molecular engineering of the keratinase from *Brevibacillus parabrevis* for dehairing performance. J. Biotechnol..

[52] Iván G., Szabadka Z., Ördög R., Grolmusz V., Náray-Szabó G. (2009). Four spatial points that define enzyme families. Biochem. Biophys. Res. Commun..

[53] Barrett A.J., Rawlings N.D. (2007). “Species” of peptidases. Biol. Chem..

[54] Betzel C., Rawlings N., Salvesen G. (2013). Thermitase. Handbook of Proteolytic Enzymes.

[55] Gupta R., Ramnani P. (2006). Microbial keratinases and their prospective applications: An overview. Appl. Microbiol. Biotechnol..

[56] Gegeckas A., Gudiukaite R., Citavicius D. (2014). Keratinolytic proteinase from *Bacillus thuringiensis* AD-12. Int. J. Biol. Macromol..

[57] Lin X., Lee C.G., Casale E.S., Shih J.C.H. (1992). Purification and characterization of a keratinase from a feather-degrading *Bacillus licheniformis* strain. Appl. Environ. Microbiol..

[58] Banerjee A., Sahoo D.K., Thatoi H., Pati B.R., Mondal K.C., Sen A., Mohapatra P.K. (2014). Structural characterization and active site prediction of bacterial keratinase through molecular docking. J. Bioinform..

[59] Guruprasad K., Reddy B.V., Pandit M.W. (1990). Correlation between stability of a protein and its dipeptide composition: A novel approach for predicting in vivo stability of a protein from its primary sequence. Protein Eng. Des. Sel..

[60] Dutta B., Banerjee A., Chakraborty P., Bandopadhyay R. (2018). In silico studies on bacterial xylanase enzyme: Structural and functional insight. J. Genet. Eng. Biotechnol..

[61] Ikai A. (1980). Thermostability and aliphatic index of globular proteins. J. Biochem..

[62] Nnolim N.E., Mpaka L., Okoh A.I., Nwodo U.U. (2020). Biochemical and molecular characterization of a thermostable alkaline metallo-keratinase from *Bacillus* sp. Nnolim-K1. Microorganisms.

[63] Gupta S., Tewatia P., Misri J., Singh R. (2017). Molecular modeling of cloned *Bacillus subtilis* keratinase and its insinuation in psoriasis treatment using docking studies. Indian J. Microbiol..

[64] Kandasamy S., Duraisamy S., Chinnappan S., Balakrishnan S., Thangasamy S., Muthusamy G., Arumugam S., Palanisamy S. (2018). Molecular modeling and docking of protease from *Bacillus* Sp. for the keratin degradation. Biocatal. Agric. Biotechnol..

