# Supplementary material for: Novel Feather Degrading Keratinases from Bacillus cereus Group: Biochemical, Genetic and Bioinformatics Analysis
Source: Microorganisms. 2022 Jan 1;10(1):93. doi: 10.3390/microorganisms10010093 (PMC8781890; doi:10.3390/microorganisms10010093)
Supplement: Supplementary file 1 [file microorganisms-10-00093-s001.zip › Figure S1.pdf]

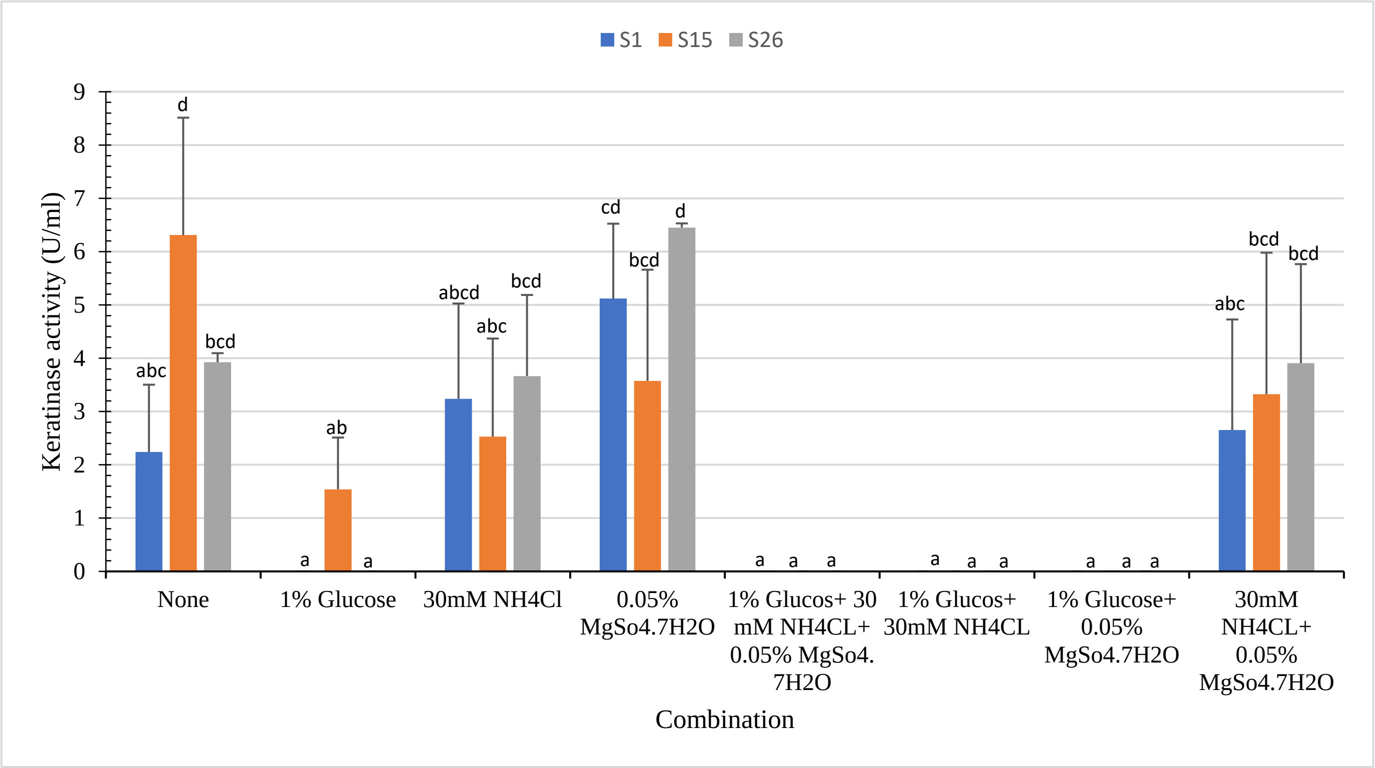

Figure S1. Effect of Glucose, NH_4CL, and $MgSO_4.7H_2O$ individually and in combination on keratinase activity by isolates S1, S15, and S26.
