# Supplementary material for: Novel Feather Degrading Keratinases from Bacillus cereus Group: Biochemical, Genetic and Bioinformatics Analysis
Source: Microorganisms. 2022 Jan 1;10(1):93. doi: 10.3390/microorganisms10010093 (PMC8781890; doi:10.3390/microorganisms10010093)
Supplement: Supplementary file 1 [file microorganisms-10-00093-s001.zip › Figure S2.pdf]

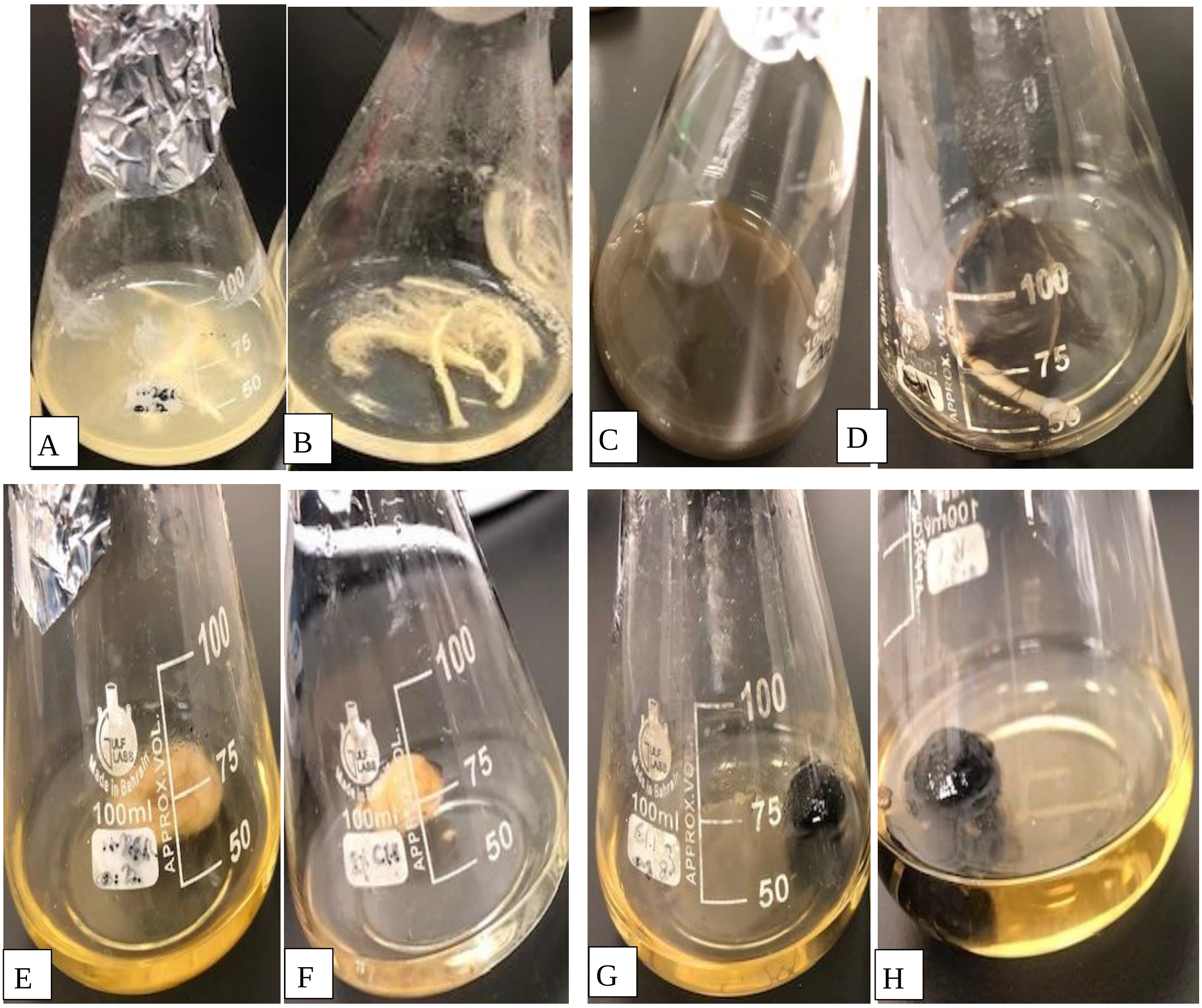

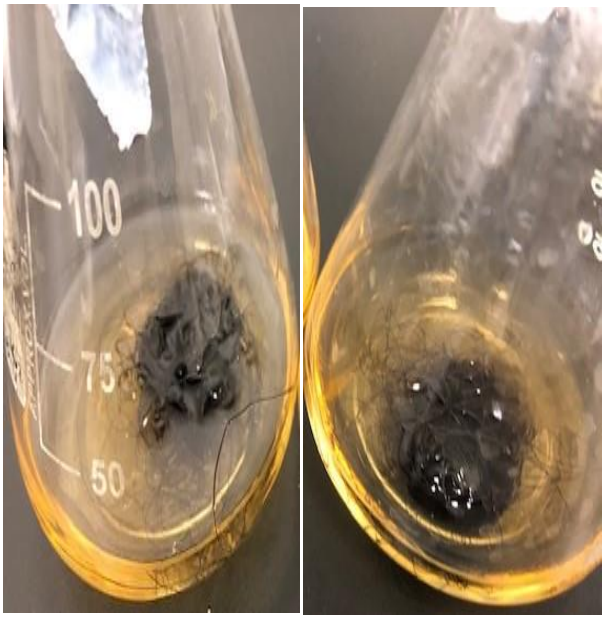

Figure S2. Influence of different keratin substrate (white chicken feather, black chicken feather, white sheep wool, black sheep wool and human hair) on keratinase production and chicken feather degradation after 72h incubation at 45 °C (A) Degradation of white chicken feather, (B) control, (C) degradation of black chicken feather, (D) control, (E) degradation of white sheep, (F) control, (G) degradation of black sheep wool, (H) control, (I) degradation of human hair and (J) control.
