# Supplementary material for: Novel Feather Degrading Keratinases from Bacillus cereus Group: Biochemical, Genetic and Bioinformatics Analysis
Source: Microorganisms. 2022 Jan 1;10(1):93. doi: 10.3390/microorganisms10010093 (PMC8781890; doi:10.3390/microorganisms10010093)
Supplement: Supplementary file 1 [file microorganisms-10-00093-s001.zip › Figure S3.pdf]

```
                                         10        20        30        40        50        60        70        80        90       100
                                ....|....|....|....|....|....|....|....|....|....|....|....|....|....|....|....|....|....|....|....|
S.26                            GGCGGACGGGTGAGTAACACGTGGGTAACCTGCCCATAAGACTGGGATAACTCCGGGAAACCGGGGCTAATACCGGATAACATTTTGAACCGCATGGTTC
CP063158.1:Bacillus_cereus      ....................................................................................................
CP072774.1:Bacillus_cereus      ....................................................................................................
CP053972.1:Bacillus_thuringien  ..........................................................................................T.........
CP051858.1:Bacillus_thuringien  ..........................................................................................T.........
MT372153.1:Bacillus_manliponen  ....................................................................................................
MW250250.1:Bacillus_pacificus   ....................................................................................................
MW115619.1:Bacillus_paramycoid  ....................................................................................................
LC178546.1:Bacillus_subtilis    ..................................TG..........................................GGTTG.................
CP050532.1:Bacillus_subtilis    ..................................TG..........................................GGTTG.................
CP026662.1:Bacillus_subtilis    ..................................TG..........................................GGTTG.................
CP049741.1:Bacillus_velezensis  ..................................TG..........................................GGTTG.................
AB592329.1:Bacillus_lichenifor  ..................................TG..........................................GCTTGA................
MK855401.1:Bacillus_lichenifor  ..................................TG..........................................GCTTGA................
CP045814.1:Bacillus_lichenifor  ..................................TG..........................................GCTTGA................
CP042252.1:Bacillus_lichenifor  ..................................TG..........................................GCTTGA................
LR698983.1:Bacillus_lichenifor  ..................................TG..........................................GCTTGA................
AB680855.1:Bacillus_atrophaeus  ..................................TG..........................................GCTTG.................
CP002207.1:Bacillus_atrophaeus  ..................................TG..........................................GCTTG.................
CP007640.1:Bacillus_atrophaeus  ..................................TG..........................................GCTTG.................

                                        110       120       130       140       150       160       170       180       190       200
                                ....|....|....|....|....|....|....|....|....|....|....|....|....|....|....|....|....|....|....|....|
S.26                            GAAAT-TGAAAGGCGGCTTC-GGCTGTCACTTATGGATGGACCCGCGTCGCATTAGCTAGTTGGTGAGGTAACGGCTCACCAAGGCAACGATGCGTAGCC
CP063158.1:Bacillus_cereus      .....-..............-...............................................................................
CP072774.1:Bacillus_cereus      .....-..............-...............................................................................
CP053972.1:Bacillus_thuringien  .....-..............-...............................................................................
CP051858.1:Bacillus_thuringien  .....-..............-...............................................................................
MT372153.1:Bacillus_manliponen  .....-..............-...............................................................................
MW250250.1:Bacillus_pacificus   .....-..............-...............................................................................
MW115619.1:Bacillus_paramycoid  ....................-...............................................................................
LC178546.1:Bacillus_subtilis    -...CA.A.....T......-....AC......CA............G....................................................
CP050532.1:Bacillus_subtilis    -...CA.A..-..T......-....AC.....GCA............G........................T...........................
CP026662.1:Bacillus_subtilis    -...CA.A.....T......-....AC......CA............G....................................................
CP049741.1:Bacillus_velezensis  -.G.CA.A.....T......-....AC......CA............G......................................G.............
AB592329.1:Bacillus_lichenifor  -..TCA.A.....T.....TTA...AC......CA............G......................................G.............
MK855401.1:Bacillus_lichenifor  -..TCA.A.....T.....TTA...AC......CA............G......................................G.............
CP045814.1:Bacillus_lichenifor  -..TCA.A.....T.....TTA...AC......CA............G......................................G.............
CP042252.1:Bacillus_lichenifor  -..TCA.A.....T.....TTA...AC......CA............G......................................G.............
LR698983.1:Bacillus_lichenifor  -..TCA.A.....T.....TTA...AC......CA............G......................................G.............
AB680855.1:Bacillus_atrophaeus  -...CA.A.....T......-....AC......CA............G....................................................
CP002207.1:Bacillus_atrophaeus  -...CA.A.....T......-....AC......CA............G........................T...........................
```

```
CP007640.1:Bacillus_atrophaeus   -...CA.......T......-....AC......CA............G........................T...........................

                                          210       220       230       240       250       260       270       280       290       300
                                 ....|....|....|....|....|....|....|....|....|....|....|....|....|....|....|....|....|....|....|....|
S.26                             GACCTGAGAGGGTGATCGGCCACACTGGGACTGAGACACGGCCCAGACTCCTACGGGAGGCAGCAGTAGGGAATCTTCCGCAATGGACGAAAGTCTGACG
CP063158.1:Bacillus_cereus       ....................................................................................................
CP072774.1:Bacillus_cereus       ....................................................................................................
CP053972.1:Bacillus_thuringien   ....................................................................................................
CP051858.1:Bacillus_thuringien   ....................................................................................................
MT372153.1:Bacillus_manliponen   ....................................................................................................
MW250250.1:Bacillus_pacificus    ....................................................................................................
MW115619.1:Bacillus_paramycoid   ....................................................................................................
LC178546.1:Bacillus_subtilis     ....................................................................................................
CP050532.1:Bacillus_subtilis     ....................................................................................................
CP026662.1:Bacillus_subtilis     ....................................................................................................
CP049741.1:Bacillus_velezensis   ....................................................................................................
AB592329.1:Bacillus_lichenifor   ............C..-....................................................................................
MK855401.1:Bacillus_lichenifor   ....................................................................................................
CP045814.1:Bacillus_lichenifor   ....................................................................................................
CP042252.1:Bacillus_lichenifor   ....................................................................................................
LR698983.1:Bacillus_lichenifor   ....................................................................................................
AB680855.1:Bacillus_atrophaeus   ....................................................................................................
CP002207.1:Bacillus_atrophaeus   ....................................................................................................
CP007640.1:Bacillus_atrophaeus   ....................................................................................................

                                          310       320       330       340       350       360       370       380       390       400
                                 ....|....|....|....|....|....|....|....|....|....|....|....|....|....|....|....|....|....|....|....|
S.26                             GAGCAACGCCGCGTGAGTGATGAAGGCTTTCGGGTCGTAAAACTCTGTTGTTAGGGAAGAACAAGTGCTAGTTGAATAAGCTGGCACCTTGACGGTACCT
CP063158.1:Bacillus_cereus       ....................................................................................................
CP072774.1:Bacillus_cereus       ....................................................................................................
CP053972.1:Bacillus_thuringien   ....................................................................................................
CP051858.1:Bacillus_thuringien   ....................................................................................................
MT372153.1:Bacillus_manliponen   ....................................................................................................
MW250250.1:Bacillus_pacificus    ....................................................................................................
MW115619.1:Bacillus_paramycoid   ....................................................................................................
LC178546.1:Bacillus_subtilis     ..........................T......A.......G........................A.CGT.C.....G.GC..T...............
CP050532.1:Bacillus_subtilis     ..........................T......A.......G..........................CGT.C.....G.GC..T...............
CP026662.1:Bacillus_subtilis     ..........................T......A.......G........................A.CGT.C.....G.GC..T...............
CP049741.1:Bacillus_velezensis   ..........................T......A.......G..........................CGT.CA....G.GC..................
AB592329.1:Bacillus_lichenifor   ..........................T......A................................A.CGT.C.....G.GC..T...............
MK855401.1:Bacillus_lichenifor   ..........................T......A................................A.CGT.C.....G.GC..T...............
CP045814.1:Bacillus_lichenifor   ..........................T......A................................A.CGT.C.....G.GC..T...............
CP042252.1:Bacillus_lichenifor   ..........................T......A................................A.CGT.C.....G.GC..T...............
LR698983.1:Bacillus_lichenifor   ..........................T......A................................A.CGT.C.....G.GC..T...............
AB680855.1:Bacillus_atrophaeus   ..........................T......A.......G..........................CGT.CA....G.GC..................
CP002207.1:Bacillus_atrophaeus   ..........................T......A.......G..........................CGT.CA....G.GC..................
```

```
CP007640.1:Bacillus_atrophaeus      ..........................T......A.......G..........................CGT.CA....G.GC..................

                                           410       420       430       440       450       460       470       480       490       500
                                    ....|....|....|....|....|....|....|....|....|....|....|....|....|....|....|....|....|....|....|....|
S.26                                AACCAGAAAGCCACGGCTAACTACGTGCCAGCAGCCGCGGTAATACGTAGGTGGCAAGCGTTATCCGGAATTATTGGGCGTAAAGCGCGCGCAGGTGGTT
CP063158.1:Bacillus_cereus          ....................................................................................................
CP072774.1:Bacillus_cereus          ....................................................................................................
CP053972.1:Bacillus_thuringien      ....................................................................................................
CP051858.1:Bacillus_thuringien      ....................................................................................................
MT372153.1:Bacillus_manliponen      ....................................................................................................
MW250250.1:Bacillus_pacificus       ....................................................................................................
MW115619.1:Bacillus_paramycoid      ..............................................................★................................★....
LC178546.1:Bacillus_subtilis        ..............................................................G......................G..T......C....
CP050532.1:Bacillus_subtilis        ..............................................................G......................G..T......C....
CP026662.1:Bacillus_subtilis        ..............................................................G......................G..T......C....
CP049741.1:Bacillus_velezensis      ..............................................................G......................G..T......C....
AB592329.1:Bacillus_lichenifor      ..............................................................G................................C....
MK855401.1:Bacillus_lichenifor      ..............................................................G................................C....
CP045814.1:Bacillus_lichenifor      ..............................................................G................................C....
CP042252.1:Bacillus_lichenifor      ..............................................................G................................C....
LR698983.1:Bacillus_lichenifor      ..............................................................G................................C....
AB680855.1:Bacillus_atrophaeus      ..............................................................G......................G..T......C....
CP002207.1:Bacillus_atrophaeus      ..............................................................G......................G..T......C....
CP007640.1:Bacillus_atrophaeus      ..............................................................G......................G..T......C....

                                           510       520       530       540       550       560       570       580       590       600
                                    ....|....|....|....|....|....|....|....|....|....|....|....|....|....|....|....|....|....|....|....|
S.26                                TCTTAAGTCTGATGTGAAAGCCCACGGCTCAACCGTGGAGGGTCATTGGAAACTGGGAGACTTGAGTGCAGAAGAGGAAAGTGGAATTCCATGTGTAGCG
CP063158.1:Bacillus_cereus          ....................................................................................................
CP072774.1:Bacillus_cereus          ....................................................................................................
CP053972.1:Bacillus_thuringien      ....................................................................................................
CP051858.1:Bacillus_thuringien      ....................................................................................................
MT372153.1:Bacillus_manliponen      ....................................................................................................
MW250250.1:Bacillus_pacificus       ...................... ..............................................................................
MW115619.1:Bacillus_paramycoid      .......................★...........★.....................★★...................★............★........
LC178546.1:Bacillus_subtilis        .......................C...........G.....................GA...................G............C........
CP050532.1:Bacillus_subtilis        .......................C...........G.....................GA...................G............C........
CP026662.1:Bacillus_subtilis        .......................C...........G.....................GA...................G............C........
CP049741.1:Bacillus_velezensis      .......................C...........G.....................GA...................G............C........
AB592329.1:Bacillus_lichenifor      .......................C...........G.....................GA........... ........G............C........
MK855401.1:Bacillus_lichenifor      .......................C...........G.....................GA...................G............C........
CP045814.1:Bacillus_lichenifor      .......................C...........G.....................GA...................G............C........
CP042252.1:Bacillus_lichenifor      .......................C...........G.....................GA...................G............C........
LR698983.1:Bacillus_lichenifor      .......................C...........G.....................GA...................G............C........
AB680855.1:Bacillus_atrophaeus      .......................C...........G.....................GA...................G............C........
CP002207.1:Bacillus_atrophaeus      .......................C...........G.....................GA...................G............C........
```

```
CP007640.1:Bacillus_atrophaeus  .......................C...........G.....................GA...................G............C........

                                      610       620       630       640       650       660       670       680       690       700
                                ....|....|....|....|....|....|....|....|....|....|....|....|....|....|....|....|....|....|....|....|
S.26                            GTGAAATGCGTAGAGATATGGAGGAACACCAGTGGCGAAGGCGACTTTCTGGTCTGTAACTGACACTGAGGCGCGAAAGCGTGGGGAGCAAACAGGATTA
CP063158.1:Bacillus_cereus      ....................................................................................................
CP072774.1:Bacillus_cereus      ....................................................................................................
CP053972.1:Bacillus_thuringien  ....................................................................................................
CP051858.1:Bacillus_thuringien  ....................................................................................................
MT372153.1:Bacillus_manliponen  ....................................................................................................
MW250250.1:Bacillus_pacificus   ....................................................................................................
MW115619.1:Bacillus_paramycoid  .................★............................★.................★........................★..........
LC178546.1:Bacillus_subtilis    .................G............................C.................G......A.................G..........
CP050532.1:Bacillus_subtilis    .................G............................C.................G......A.................G..........
CP026662.1:Bacillus_subtilis    .................G............................C.................G......A.................G..........
CP049741.1:Bacillus_velezensis  .................G............................C.................G......A.................G..........
AB592329.1:Bacillus_lichenifor  ............A....G............................C.................G........................G..........
MK855401.1:Bacillus_lichenifor  .................G............................C.................G........................G..........
CP045814.1:Bacillus_lichenifor  .................G............................C.................G........................G..........
CP042252.1:Bacillus_lichenifor  .................G............................C.................G........................G..........
LR698983.1:Bacillus_lichenifor  .................G............................C.................G........................G..........
AB680855.1:Bacillus_atrophaeus  .................G............................C.................G......A.................G..........
CP002207.1:Bacillus_atrophaeus  .................G............................C.................G......A.................G..........
CP007640.1:Bacillus_atrophaeus  .................G............................C.................G......A.................G..........

                                      710       720       730       740       750       760       770       780       790       800
                                ....|....|....|....|....|....|....|....|....|....|....|....|....|....|....|....|....|....|....|....|
S.26                            GATACCCTGGTAGTCCACGCCGTAAACGATGAGTGCTAAGTGTTAGAGGGTTTCCGCCCTTTAGTGCTGAAGTTAACGCATTAAGCACTCCGCCTGGGGA
CP063158.1:Bacillus_cereus      ....................................................................................................
CP072774.1:Bacillus_cereus      ....................................................................................................
CP053972.1:Bacillus_thuringien  ....................................................................................................
CP051858.1:Bacillus_thuringien  ....................................................................................................
MT372153.1:Bacillus_manliponen  ....................................................................................................
MW250250.1:Bacillus_pacificus   ....................................................................................................
MW115619.1:Bacillus_paramycoid  .....................................................................★..★...........................
LC178546.1:Bacillus_subtilis    ..............................................G............C.........C..C...........................
CP050532.1:Bacillus_subtilis    ..............................................G............C.........C..C...........................
CP026662.1:Bacillus_subtilis    ..............................................G............C.........C..C...........................
CP049741.1:Bacillus_velezensis  ..............................................G............C.........C..C...........................
AB592329.1:Bacillus_lichenifor  .....................................................................C..CA..........................
MK855401.1:Bacillus_lichenifor  .....................................................................C..CA..........................
CP045814.1:Bacillus_lichenifor  .....................................................................C..CA..........................
CP042252.1:Bacillus_lichenifor  .....................................................................C..CA..........................
LR698983.1:Bacillus_lichenifor  .....................................................................C..CA..........................
AB680855.1:Bacillus_atrophaeus  ..............................................G............C.........C..C...........................
CP002207.1:Bacillus_atrophaeus  ..............................................G............C.........C..C...........................
```

```
CP007640.1:Bacillus_atrophaeus   ..............................................G............C.........C..C...........................

                                          810       820       830       840       850       860       870       880       890       900
                                 ....|....|....|....|....|....|....|....|....|....|....|....|....|....|....|....|....|....|....|....|
S.26                             GTACGGCCGCAAGGCTGAAACTCAAAGGAATTGACGGGGGCCCGCACAAGCGGTGGAGCATGTGGTTTAATTCGAAGCAACGCGAAGAACCTTACCAGGT
CP063158.1:Bacillus_cereus       ....................................................................................................
CP072774.1:Bacillus_cereus       ....................................................................................................
CP053972.1:Bacillus_thuringien   ....................................................................................................
CP051858.1:Bacillus_thuringien   ....................................................................................................
MT372153.1:Bacillus_manliponen   ....................................................................................................
MW250250.1:Bacillus_pacificus    ....................................................................................................
MW115619.1:Bacillus_paramycoid   ......★......★......................................................................................
LC178546.1:Bacillus_subtilis     ......T......A......................................................................................
CP050532.1:Bacillus_subtilis     ......T......A......................................................................................
CP026662.1:Bacillus_subtilis     ......T......A......................................................................................
CP049741.1:Bacillus_velezensis   ......T......A......................................................................................
AB592329.1:Bacillus_lichenifor   ......T......A......................................................................................
MK855401.1:Bacillus_lichenifor   ......T......A......................................................................................
CP045814.1:Bacillus_lichenifor   ......T......A......................................................................................
CP042252.1:Bacillus_lichenifor   ......T......A......................................................................................
LR698983.1:Bacillus_lichenifor   ......T......A......................................................................................
AB680855.1:Bacillus_atrophaeus   ......T......A......................................................................................
CP002207.1:Bacillus_atrophaeus   ......T......A......................................................................................
CP007640.1:Bacillus_atrophaeus   ......T......A......................................................................................

                                          910       920       930       940       950       960       970       980       990      1000
                                 ....|....|....|....|....|....|....|....|....|....|....|....|....|....|....|....|....|....|....|....|
S.26                             CTTGACATCCTCTGAAAACCCTAGAGATAGGGCTTCTCCTTCGGGAGCAGAGTGACAGGTGGTGCATGGTTGTCGTCAGCTCGTGTCGTGAGATGTTGGG
CP063158.1:Bacillus_cereus       ....................................................................................................
CP072774.1:Bacillus_cereus       ....................................................................................................
CP053972.1:Bacillus_thuringien   ....................................................................................................
CP051858.1:Bacillus_thuringien   ....................................................................................................
MT372153.1:Bacillus_manliponen   ....................................................................................................
MW250250.1:Bacillus_pacificus    ....................................................................................................
MW115619.1:Bacillus_paramycoid   ...............★....................★........★......................................................
LC178546.1:Bacillus_subtilis     ...............C..T............A.G..C........G......................................................
CP050532.1:Bacillus_subtilis     ...............C..T............A.G..C........G......................................................
CP026662.1:Bacillus_subtilis     ...............C..T............A.G..C........G......................................................
CP049741.1:Bacillus_velezensis   ...............C..T............A.G..C........G......................................................
AB592329.1:Bacillus_lichenifor   ...............C....................C........G......................................................
MK855401.1:Bacillus_lichenifor   ...............C....................C........G......................................................
CP045814.1:Bacillus_lichenifor   ...............C....................C........G......................................................
CP042252.1:Bacillus_lichenifor   ...............C....................C........G......................................................
LR698983.1:Bacillus_lichenifor   ...............C....................C........G......................................................
AB680855.1:Bacillus_atrophaeus   ...............C.C..................C........G......................................................
CP002207.1:Bacillus_atrophaeus   ...............C.C..................C........G......................................................
```

```
CP007640.1:Bacillus_atrophaeus   ...............C.C..................C........G......................................................

                                          1010      1020      1030      1040      1050      1060      1070      1080      1090      1100
                                 ....|....|....|....|....|....|....|....|....|....|....|....|....|....|....|....|....|....|....|....|
S.26                             TTAAGTCCCGCAACGAGCGCAACCCTTGATCTTAGTTGCCATCATTAAGTTGGGCACTCTAAGGTGACTGCCGGTGACAAACCGGAGGAAGGTGGGGATG
CP063158.1:Bacillus_cereus       ....................................................................................................
CP072774.1:Bacillus_cereus       ....................................................................................................
CP053972.1:Bacillus_thuringien   ....................................................................................................
CP051858.1:Bacillus_thuringien   ....................................................................................................
MT372153.1:Bacillus_manliponen   ....................................................................................................
MW250250.1:Bacillus_pacificus    ....................................................................................................
MW115619.1:Bacillus_paramycoid   .........................................★....★.....................................................
LC178546.1:Bacillus_subtilis     .........................................G....C.....................................................
CP050532.1:Bacillus_subtilis     .........................................G....C.....................................................
CP026662.1:Bacillus_subtilis     .........................................G....C............... ......................................
CP049741.1:Bacillus_velezensis   .........................................G....C.....................................................
AB592329.1:Bacillus_lichenifor   .........................................G....C.....................................................
MK855401.1:Bacillus_lichenifor   .........................................G....C.....................................................
CP045814.1:Bacillus_lichenifor   .........................................G....C.....................................................
CP042252.1:Bacillus_lichenifor   .........................................G....C.....................................................
LR698983.1:Bacillus_lichenifor   .........................................G....C.....................................................
AB680855.1:Bacillus_atrophaeus   .........................................G....C.....................................................
CP002207.1:Bacillus_atrophaeus   .........................................G....C.....................................................
CP007640.1:Bacillus_atrophaeus   .........................................G....C.....................................................

                                          1110      1120      1130      1140      1150      1160      1170      1180      1190      1200
                                 ....|....|....|....|....|....|....|....|....|....|....|....|....|....|....|....|....|....|....|....|
S.26                             ACGTCAAATCATCATGCCCCTTATGACCTGGGCTACACACGTGCTACAATGGACGGTACAAAGAGCTGCAAGACCGCGAGGTGGAGCTAATCTCATAAAA
CP063158.1:Bacillus_cereus       ....................................................................................................
CP072774.1:Bacillus_cereus       ....................................................................................................
CP053972.1:Bacillus_thuringien   ....................................................................................................
CP051858.1:Bacillus_thuringien   ....................................................................................................
MT372153.1:Bacillus_manliponen   ....................................................................................................
MW250250.1:Bacillus_pacificus    ....................................................................................................
MW115619.1:Bacillus_paramycoid   ......................................................★.★......★..★..★............★★...★....★..★...★
LC178546.1:Bacillus_subtilis     ......................................................A.A......G..A..G.A..........TA...C....C..C...T
CP050532.1:Bacillus_subtilis     ......................................................A.A......G..A..G.A..........TA...C....C..C...T
CP026662.1:Bacillus_subtilis     ......................................................A.A......G..A..G.A..........TA...C....C..C...T
CP049741.1:Bacillus_velezensis   ......................................................A.A......G..A..G.A..........TA...C....C..C...T
AB592329.1:Bacillus_lichenifor   ....................................................G.A.A......G..A..G.AG........CTA...C....C..C...T
MK855401.1:Bacillus_lichenifor   ....................................................G.A.A......G..A..G.AG........CTA...C....C..C...T
CP045814.1:Bacillus_lichenifor   ....................................................G.A.A......G..A..G.AG........CTA...C....C..C...T
CP042252.1:Bacillus_lichenifor   ....................................................G.A.A......G..A..G.AG........CTA...C. ...C..C...T
LR698983.1:Bacillus_lichenifor   ....................................................G.A.A......G..A..G.AG........CTA...C....C..C...T
AB680855.1:Bacillus_atrophaeus   ......................................................A.A......G..A..G............TA...C....C..C...T
CP002207.1:Bacillus_atrophaeus   ......................................................A.A......G..A..G............TA...C....C..C...T
```

```
CP007640.1:Bacillus_atrophaeus  ......................................................A.A......G..A..G............TA...C....C..C...T



                                ....|....
S.26                            CCGTTCTCA
CP063158.1:Bacillus_cereus      .........
CP072774.1:Bacillus_cereus      .........
CP053972.1:Bacillus_thuringien  .........
CP051858.1:Bacillus_thuringien  .........
MT372153.1:Bacillus_manliponen  .........
MW250250.1:Bacillus_pacificus   .........
                                 ★
MW115619.1:Bacillus_paramycoid  .........
LC178546.1:Bacillus_subtilis    .T.......
CP050532.1:Bacillus_subtilis    .T.......
CP026662.1:Bacillus_subtilis    .T.......
CP049741.1:Bacillus_velezensis  .T.......
AB592329.1:Bacillus_lichenifor  .T.......
MK855401.1:Bacillus_lichenifor  .T.......
CP045814.1:Bacillus_lichenifor  .T.......
CP042252.1:Bacillus_lichenifor  .T.......
LR698983.1:Bacillus_lichenifor  .T.......
AB680855.1:Bacillus_atrophaeus  .T.......
CP002207.1:Bacillus_atrophaeus  .T.......
CP007640.1:Bacillus_atrophaeus  .T.......
```

Figure S3. Multiple sequence analysis of *16S rRNA* gene, strain S26 against sequences retrieved from GenBank database. Stars correspond to the 58 major substitution position differentiated between *B. subtilis* and *B. cereus* groups. Strain S26 is identical to *B. cereus* groups sequences.
