# Supplementary material for: Novel Feather Degrading Keratinases from Bacillus cereus Group: Biochemical, Genetic and Bioinformatics Analysis
Source: Microorganisms. 2022 Jan 1;10(1):93. doi: 10.3390/microorganisms10010093 (PMC8781890; doi:10.3390/microorganisms10010093)
Supplement: Supplementary file 1 [file microorganisms-10-00093-s001.zip › Figure S4.pdf]

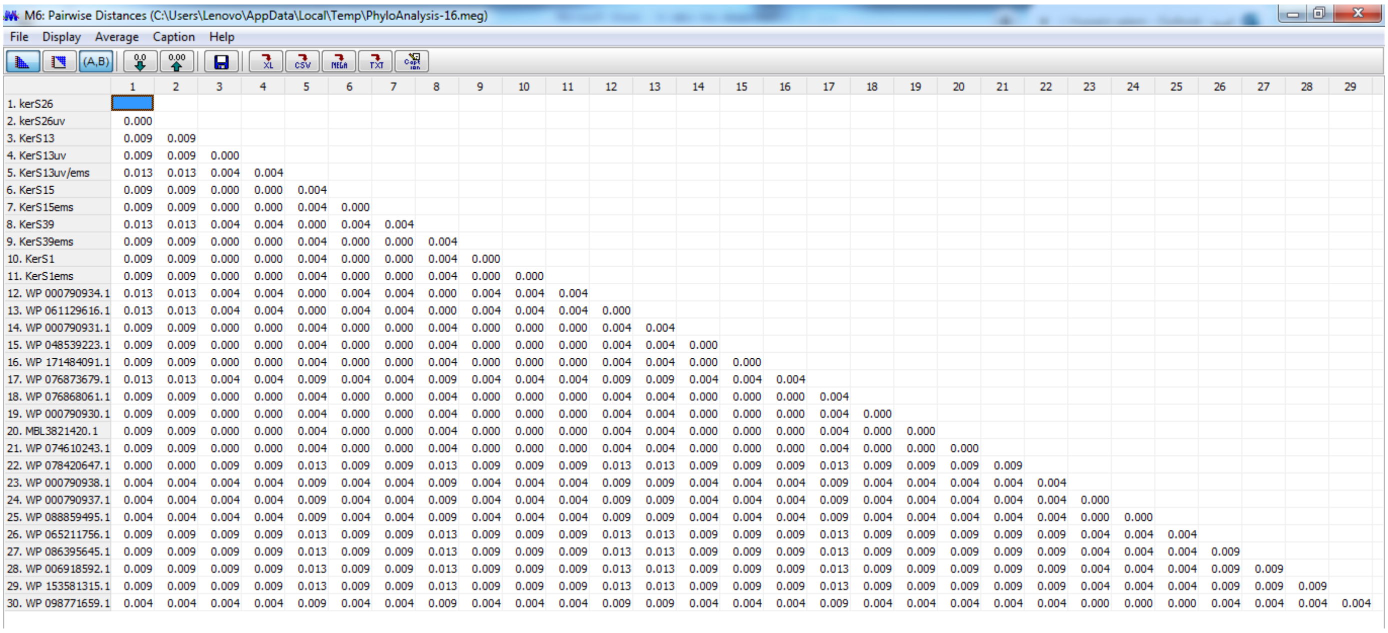


| | 1 | 2 | 3 | 4 | 5 | 6 | 7 | 8 | 9 | 10 | 11 | 12 | 13 | 14 | 15 | 16 | 17 | 18 | 19 | 20 | 21 | 22 | 23 | 24 | 25 | 26 | 27 | 28 | 29 |
|---|---|---|---|---|---|---|---|---|---|---|---|---|---|---|---|---|---|---|---|---|---|---|---|---|---|---|---|---|---|
| 1. kerS26 | | | | | | | | | | | | | | | | | | | | | | | | | | | | | |
| 2. kerS26uv | 0.000 | | | | | | | | | | | | | | | | | | | | | | | | | | | | |
| 3. KerS13 | 0.009 | 0.009 | | | | | | | | | | | | | | | | | | | | | | | | | | | |
| 4. KerS13uv | 0.009 | 0.009 | 0.000 | | | | | | | | | | | | | | | | | | | | | | | | | | |
| 5. KerS13uv/ems | 0.013 | 0.013 | 0.004 | 0.004 | | | | | | | | | | | | | | | | | | | | | | | | | |
| 6. KerS15 | 0.009 | 0.009 | 0.000 | 0.000 | 0.004 | | | | | | | | | | | | | | | | | | | | | | | | |
| 7. KerS15ems | 0.009 | 0.009 | 0.000 | 0.000 | 0.004 | 0.000 | | | | | | | | | | | | | | | | | | | | | | | |
| 8. KerS39 | 0.013 | 0.013 | 0.004 | 0.004 | 0.000 | 0.004 | 0.004 | | | | | | | | | | | | | | | | | | | | | | |
| 9. KerS39ems | 0.009 | 0.009 | 0.000 | 0.000 | 0.004 | 0.000 | 0.000 | 0.004 | | | | | | | | | | | | | | | | | | | | | |
| 10. KerS1 | 0.009 | 0.009 | 0.000 | 0.000 | 0.004 | 0.000 | 0.000 | 0.004 | 0.000 | | | | | | | | | | | | | | | | | | | | |
| 11. KerS1ems | 0.009 | 0.009 | 0.000 | 0.000 | 0.004 | 0.000 | 0.000 | 0.004 | 0.000 | 0.000 | | | | | | | | | | | | | | | | | | | |
| 12. WP 000790934.1 | 0.013 | 0.013 | 0.004 | 0.004 | 0.000 | 0.004 | 0.004 | 0.000 | 0.004 | 0.004 | 0.004 | | | | | | | | | | | | | | | | | | |
| 13. WP 061129616.1 | 0.013 | 0.013 | 0.004 | 0.004 | 0.000 | 0.004 | 0.004 | 0.000 | 0.004 | 0.004 | 0.004 | 0.000 | | | | | | | | | | | | | | | | | |
| 14. WP 000790931.1 | 0.009 | 0.009 | 0.000 | 0.000 | 0.004 | 0.000 | 0.000 | 0.004 | 0.000 | 0.000 | 0.000 | 0.004 | 0.004 | | | | | | | | | | | | | | | | |
| 15. WP 048539223.1 | 0.009 | 0.009 | 0.000 | 0.000 | 0.004 | 0.000 | 0.000 | 0.004 | 0.000 | 0.000 | 0.000 | 0.004 | 0.004 | 0.000 | | | | | | | | | | | | | | | |
| 16. WP 171484091.1 | 0.009 | 0.009 | 0.000 | 0.000 | 0.004 | 0.000 | 0.000 | 0.004 | 0.000 | 0.000 | 0.000 | 0.004 | 0.004 | 0.000 | 0.000 | | | | | | | | | | | | | | |
| 17. WP 076873679.1 | 0.013 | 0.013 | 0.004 | 0.004 | 0.009 | 0.004 | 0.004 | 0.009 | 0.004 | 0.004 | 0.004 | 0.009 | 0.009 | 0.004 | 0.004 | 0.004 | | | | | | | | | | | | | |
| 18. WP 076868061.1 | 0.009 | 0.009 | 0.000 | 0.000 | 0.004 | 0.000 | 0.000 | 0.004 | 0.000 | 0.000 | 0.000 | 0.004 | 0.004 | 0.000 | 0.000 | 0.000 | 0.004 | | | | | | | | | | | | |
| 19. WP 000790930.1 | 0.009 | 0.009 | 0.000 | 0.000 | 0.004 | 0.000 | 0.000 | 0.004 | 0.000 | 0.000 | 0.000 | 0.004 | 0.004 | 0.000 | 0.000 | 0.000 | 0.004 | 0.000 | | | | | | | | | | | |
| 20. MBL3821420.1 | 0.009 | 0.009 | 0.000 | 0.000 | 0.004 | 0.000 | 0.000 | 0.004 | 0.000 | 0.000 | 0.000 | 0.004 | 0.004 | 0.000 | 0.000 | 0.000 | 0.004 | 0.000 | 0.000 | | | | | | | | | | |
| 21. WP 074610243.1 | 0.009 | 0.009 | 0.000 | 0.000 | 0.004 | 0.000 | 0.000 | 0.004 | 0.000 | 0.000 | 0.000 | 0.004 | 0.004 | 0.000 | 0.000 | 0.000 | 0.004 | 0.000 | 0.000 | 0.000 | | | | | | | | | |
| 22. WP 078420647.1 | 0.000 | 0.000 | 0.009 | 0.009 | 0.013 | 0.009 | 0.009 | 0.013 | 0.009 | 0.009 | 0.009 | 0.013 | 0.013 | 0.009 | 0.009 | 0.009 | 0.013 | 0.009 | 0.009 | 0.009 | 0.009 | | | | | | | | |
| 23. WP 000790938.1 | 0.004 | 0.004 | 0.004 | 0.004 | 0.009 | 0.004 | 0.004 | 0.009 | 0.004 | 0.004 | 0.004 | 0.009 | 0.009 | 0.004 | 0.004 | 0.004 | 0.009 | 0.004 | 0.004 | 0.004 | 0.004 | 0.004 | | | | | | | |
| 24. WP 000790937.1 | 0.004 | 0.004 | 0.004 | 0.004 | 0.009 | 0.004 | 0.004 | 0.009 | 0.004 | 0.004 | 0.004 | 0.009 | 0.009 | 0.004 | 0.004 | 0.004 | 0.009 | 0.004 | 0.004 | 0.004 | 0.004 | 0.004 | 0.000 | | | | | | |
| 25. WP 088859495.1 | 0.004 | 0.004 | 0.004 | 0.004 | 0.009 | 0.004 | 0.004 | 0.009 | 0.004 | 0.004 | 0.004 | 0.009 | 0.009 | 0.004 | 0.004 | 0.004 | 0.009 | 0.004 | 0.004 | 0.004 | 0.004 | 0.004 | 0.000 | 0.000 | | | | | |
| 26. WP 065211756.1 | 0.009 | 0.009 | 0.009 | 0.009 | 0.013 | 0.009 | 0.009 | 0.013 | 0.009 | 0.009 | 0.009 | 0.013 | 0.013 | 0.009 | 0.009 | 0.009 | 0.013 | 0.009 | 0.009 | 0.009 | 0.009 | 0.009 | 0.004 | 0.004 | 0.004 | | | | |
| 27. WP 086395645.1 | 0.009 | 0.009 | 0.009 | 0.009 | 0.013 | 0.009 | 0.009 | 0.013 | 0.009 | 0.009 | 0.009 | 0.013 | 0.013 | 0.009 | 0.009 | 0.009 | 0.013 | 0.009 | 0.009 | 0.009 | 0.009 | 0.009 | 0.004 | 0.004 | 0.004 | 0.009 | | | |
| 28. WP 006918592.1 | 0.009 | 0.009 | 0.009 | 0.009 | 0.013 | 0.009 | 0.009 | 0.013 | 0.009 | 0.009 | 0.009 | 0.013 | 0.013 | 0.009 | 0.009 | 0.009 | 0.013 | 0.009 | 0.009 | 0.009 | 0.009 | 0.009 | 0.004 | 0.004 | 0.004 | 0.009 | 0.009 | | |
| 29. WP 153581315.1 | 0.009 | 0.009 | 0.009 | 0.009 | 0.013 | 0.009 | 0.009 | 0.013 | 0.009 | 0.009 | 0.009 | 0.013 | 0.013 | 0.009 | 0.009 | 0.009 | 0.013 | 0.009 | 0.009 | 0.009 | 0.009 | 0.009 | 0.004 | 0.004 | 0.004 | 0.009 | 0.009 | 0.009 | |
| 30. WP 098771659.1 | 0.004 | 0.004 | 0.004 | 0.004 | 0.009 | 0.004 | 0.004 | 0.009 | 0.004 | 0.004 | 0.004 | 0.009 | 0.009 | 0.004 | 0.004 | 0.004 | 0.009 | 0.004 | 0.004 | 0.004 | 0.004 | 0.004 | 0.000 | 0.000 | 0.000 | 0.004 | 0.004 | 0.004 | 0.004 |

**Figure S4.** Estimates of evolutionary divergence of *KerS* gene against S8 family peptidase, *Bacillus cereus* group sequences retrieved from GenBank database.
