# Supplementary material for: Novel Feather Degrading Keratinases from Bacillus cereus Group: Biochemical, Genetic and Bioinformatics Analysis
Source: Microorganisms. 2022 Jan 1;10(1):93. doi: 10.3390/microorganisms10010093 (PMC8781890; doi:10.3390/microorganisms10010093)
Supplement: Supplementary file 1 [file microorganisms-10-00093-s001.zip › Table S1.pdf]

Table S1. BLASTp analysis of keratinase *kerS* showing deduced amino acid, total score, query cover, E-value and percentage of identity.

Keratinase gene	Deduced amino acid	Total score	Query cover	E-value	% of identity
***KerS*1**	234	(481-483)	100%	(1e-168-4e-170)	(99.15%-100%)
***KerS*1ems**	397	(795-805)	100%	0.0	(98.24%-100%)
***KerS*13**	397	(795-805)	100%	0.0	(98.24%-100%)
***KerS*13uv**	397	(795-805)	100%	0.0	(98.24%-100%)
***KerS*13uv+ems**	397	(793-804)	100%	0.0	(97.98%-100%)
***KerS*15**	397	(795-805)	100%	0.0	(98.24%-100%)
***KerS*15ems**	397	(795-805)	100%	0.0	(98.24%-100%)
***KerS*26**	397	(795-805)	100%	0.0	(98.24%-100%)
***KerS*26uv**	397	(795-805)	100%	0.0	(98.24%-100%)
***KerS*39**	295	(593-601)	(99%-100%)	0.0	(98.64%-100%)
***KerS*39ems**	269	(547-551)	100%	0.0	(99.26%-100%)
