# Supplementary material for: Novel Feather Degrading Keratinases from Bacillus cereus Group: Biochemical, Genetic and Bioinformatics Analysis
Source: Microorganisms. 2022 Jan 1;10(1):93. doi: 10.3390/microorganisms10010093 (PMC8781890; doi:10.3390/microorganisms10010093)
Supplement: Supplementary file 1 [file microorganisms-10-00093-s001.zip › Table S2.pdf]

Table S2. BLASTp analysis of *KerS* gene (*KerS*13, *KerS*13uv+ems, and *KerS*26uv) and the corresponding similar peptidase S8 *B. cereus* group species.

Isolate name	No.	Accession No.	Species	% Identity
***KerS*13**	1	WP_076873679.1	S8 family peptidase [*Bacillus cereus* group]	99.75%
	2	WP_171484091.1	S8 family serine peptidase [*Bacillus paranthracis*]	99.75%
	3	WP_088068318.1	S8 family peptidase [*Bacillus thuringiensis*]	99.24%
	4	PFJ29283.1	alkaline serine protease [*Bacillus anthracis*]	98.99%
	5	WP_144655882.1	S8 family peptidase [*Bacillus tropicus*]	98.99%
	6	WP_074563627.1	S8 family peptidase [*Bacillus cereus*]	98.74%
***KerS*13uv+ems**	1	WP_061129616.1	S8 family peptidase [*Bacillus cereus*]	99.75%
	2	WP_098852855.1	S8 family peptidase [*Bacillus thuringiensis*]	98.74%
	3	WP_197225397.1	S8 family peptidase [*Bacillus paranthracis*]	98.49%
	4	WP_098616758.1	S8 family peptidase [*Bacillus cereus group*]	98.24%
	5	MBL3821420.1	peptidase S8 [*Bacillus cereus*]	99.24%
	6	PES75482.1	alkaline serine protease [*Bacillus anthracis*]	98.24%
***KerS*26uv**	1	WP_078420647.1	S8 family peptidase [*Bacillus cereus*]	100%
	2	WP_044584435.1	S8 family peptidase [*Bacillus bombysepticus*]	99.24%
	3	WP_098356006.1	S8 family peptidase [*Bacillus thuringiensis*]	98.99%
	4	TBL05390.1	peptidase S8 [*Bacillus paranthracis*]	98.99%
	5	WP_213745030.1	S8 family serine peptidase [*Bacillus toyonensis*]	98.74%
	6	WP_099686350.1	S8 family peptidase [*Bacillus fungorum*]	98.74%
	7	WP_226638973.1	S8 family peptidase [*Bacillus tropicus*]	98.74%
	8	WP_098158349.1	S8 family peptidase [*Bacillus toyonensis*]	98.49%
